# Scoping review of social norms interventions to reduce violence and improve SRHR outcomes among adolescents and young people in sub-Saharan Africa

**DOI:** 10.3389/frph.2025.1592696

**Published:** 2025-05-15

**Authors:** Luciana Leite, Rachel Yates, Gaia Chiti Strigelli, Jenny Yi-Chen Han, Jenny Chen-Charles, Maria Rotaru, Elona Toska

**Affiliations:** ^1^ Department of Social Policy and Intervention, University of Oxford, Oxford, United Kingdom; ^2^ Centre for Social Science Research, University of Cape Town, Cape Town, South Africa; ^3^ UNICEF Eastern and Southern Africa Regional Office, (UNICEF-ESARO), Nairobi, Kenya

**Keywords:** social norms, gender norms, gender-based violence, sexual and reproductive health, interventions, scoping review, adolescents

## Abstract

**Introduction:**

Despite growing interest, guidance to inform effective social norms interventions that improve adolescents and young people's sexual and reproductive health and rights (SRHR) is needed.

**Methods:**

We conducted a scoping review of experimental and quasi-experimental studies of social norms interventions in sub-Saharan Africa. Single and multi-component interventions that included a social norms component and assessed impacts on SRHR outcomes among 10- to 24-year-old adolescents and young people were included. We mapped interventions across eight programmatic strategies and six SRHR outcomes, drawing programmatic insights.

**Results:**

*N* = 40 interventions from 12 countries reported effectiveness on intimate partner and non-partner sexual, physical and emotional violence (*N* = 14), child marriage (*N* = 6), sexual risk behaviours (*N* = 20), contraception and family planning (*N* = 23), prevention and treatment of HIV and other sexually transmitted infections (*N* = 17), and early pregnancy (*N* = 10). Intervention strategies included life skills approaches (*N* = 23), community dialogues (*N* = 14), school-based SRHR programming (*N* = 10), parenting programmes (*N* = 8), training of healthcare professionals on youth-friendly services (*N* = 7), media or digital-based approaches (*N* = 7), interventions with community leaders (*N* = 5), and rights-based advocacy (*N* = 2). Norms interventions can improve multiple SRHR outcomes, including reducing gender-based and intimate partner violence and child marriage, increasing HIV testing, and contraceptive use. Effective programmes were often implemented in combined interventions, and included life skills, community conversations, mass media and digital programmes with norms components.

**Discussion:**

Integrating gender-transformative approaches such as reflections on gender roles and inequalities, youth sexuality, and gendered power relations, and engaging with reference groups were key to the success of programmes. Effective approaches delivered SRHR information alongside reflections on social norms, and combined participatory methods with a structured curriculum guiding activities led by trained facilitators. Most interventions focused on changing social expectations and awareness, but few reported clear diffusion strategies to ensure the spread of the new norms and behaviours for the expected change. Key evidence gaps identified include integrating implementation research to inform the scale-up and sustainability of effective social norms interventions. Social norms interventions that effectively address the needs of high-risk adolescents and young people are needed.

## 1 Introduction

Adolescents and young people in sub-Saharan Africa face several barriers to reaching their full potential, including realising their sexual and reproductive health and rights. Several countries experience high rates of HIV incidence and prevalence among adolescents and young people, which disproportionately affect adolescent girls and young women who are three times as likely to acquire HIV as their male counterparts in sub-Saharan Africa (1). High rates of sexual violence, adolescent pregnancy, and early marriage further compound poor outcomes among adolescent girls and young women (2, 3). Structural barriers related to poor socio-economic conditions, such as lack of financial access and appropriate, accessible services, further heighten the risk of poor SRHR outcomes (4). Social norms, or informal, shared rules about what are acceptable and appropriate actions within a group or community, can spread harmful misinformation and lead to poor SRHR outcomes, particularly when young people also lack accurate information and knowledge to make informed SRHR decisions (5).

Gender-based violence (GBV), including intimate partner violence (IPV) and non-partner sexual violence, is a significant issue affecting adolescent girls and young women in sub-Saharan Africa (6). An estimated 33% of women aged 15–49 in the region have experienced IPV in their lifetime, with even higher rates reported in some countries (7). Violence against young women and girls can be both a consequence and a driver of poor SRHR outcomes. Experiencing violence can lead to higher risks of unintended pregnancies, unsafe abortions, HIV and STI infections, and adverse maternal health outcomes (8).

Adolescence is a time when young people establish their independence, develop behavioural patterns, including health-seeking behaviours, and experience major physical, cognitive, hormonal, and social changes (9). This can be accompanied by challenging parental and other boundaries and taking greater risks, which can make them more vulnerable to sexual exploitation. As young people reach puberty, social norms become more pronounced (10, 11). Adolescence is, therefore, a critical period to support young people as they navigate their transitions to adulthood, including by promoting positive behaviours that can bring lifelong impacts on their health and wellbeing.

Social norms, including gender norms, have been identified as critical barriers to achieving better SRHR outcomes, especially for women and girls (12, 13). Social expectations of male dominance and control can restrict girls' and young women's agency over SRHR choices and increase their vulnerability to violence. Addressing social norms that sustain gender inequality and the acceptance of violence is therefore essential to improving overall SRHR outcomes and ensuring safety and wellbeing. Whilst there is widespread recognition of the importance of norms and their application to the field of SRHR, there are significant gaps in understanding how norms impact outcomes, particularly for young people, and what works to shift social norms interventions to improve SRHR outcomes.

Adolescents and young people may lack full agency over the behaviours that determine these outcomes and need supportive environments to make informed decisions (9). These environments are shaped by reference groups, or people whose opinions and expectations matter to young people when they consider whether to engage in a specific behaviour (14). The groups of people included in young people's reference groups may vary by individual and by behaviour, but often include peers, parents or caregivers, and community members (15).

Social norms interventions to shift behaviours have been increasingly used as a strategy to promote the health and wellbeing of adolescents across sub-Saharan Africa (5). The lack of consensus on the definition of social norms programmes poses a challenge for the evaluation of their effectiveness, new designs, and scaled-up initiatives (16). Existing work to categorise strategies of norms-shifting interventions tends to focus on their behavioural change strategy (17, 18). However, these strategies may not always be as clearly defined in interventions to improve SRHR. We define social norms interventions as standalone or multi-component behaviour change interventions that seek to change the socially shared rules that determine acceptable and appropriate actions within a group or community (16, 19–21).

This review synthesises research on the effectiveness of social norms interventions to improve SRHR among adolescents and young people in sub-Saharan Africa. It focuses on evaluations of interventions' effectiveness or lack thereof in shifting social norms to improve SRHR outcomes. It seeks to assess the impact of the types of interventions on specific SRHR outcomes while highlighting insights that can be applied to social norms programming across a range of outcomes, with a deeper dive into what works to prevent violence.

## 2 Methods

We included any single or multi-component interventional study published between 2014 and 2024 that included an element aimed at norms change and assessed impacts on one or more SRHR outcomes among adolescents and young people (10–24 years old) in sub-Saharan Africa. The SRHR outcomes, selected based on consultations with key informants and UN partners, include: violence exposure including physical, sexual, and emotional forms of GBV, uptake or use of contraceptives and family planning methods, HIV incidence, testing, prevention, and treatment, STI incidence including herpes simplex virus (HSV-2), early pregnancy, sexual debut, transactional sex, age-disparate sex, multiple sexual partners, female genital mutilation/cutting and other harmful gender practices, early, forced, and child marriage, menstrual hygiene management, medical male circumcision, and abortion.

While some studies were explicit in their objective to shift social norms, we also included studies that described interventions that were implicitly aiming to bring about norms change, e.g., those addressing gender empowerment, notions of masculinity, and stigma. We included studies independent of whether they measured outcomes related to norms and attitudes, but that assessed changes in SRHR outcomes. We included quantitative evaluation studies with a control group such as randomised control trials (RCTs), and other experimental and quasi-experimental studies. Exclusively qualitative studies, or mixed methods and quantitative studies with cross-sectional and longitudinal designs, but without a control group, were not considered. Searches were conducted in five databases or registries: Web of Science, ProQuest SS Premium, PubMed, Cinahl and Cochrane, in March 2024 for peer-reviewed published studies in English. Articles in other languages were excluded. Search terms were iteratively developed within five search concepts: (1) adolescents and young people, (2) social and gender norms, (3) types of norms interventions, (4) SRHR outcomes and (5) geographical focus. The keywords and terms were combined one after the other using Boolean Operators. The search terms are further described in Supplementary Table S1.

**Figure 1:**
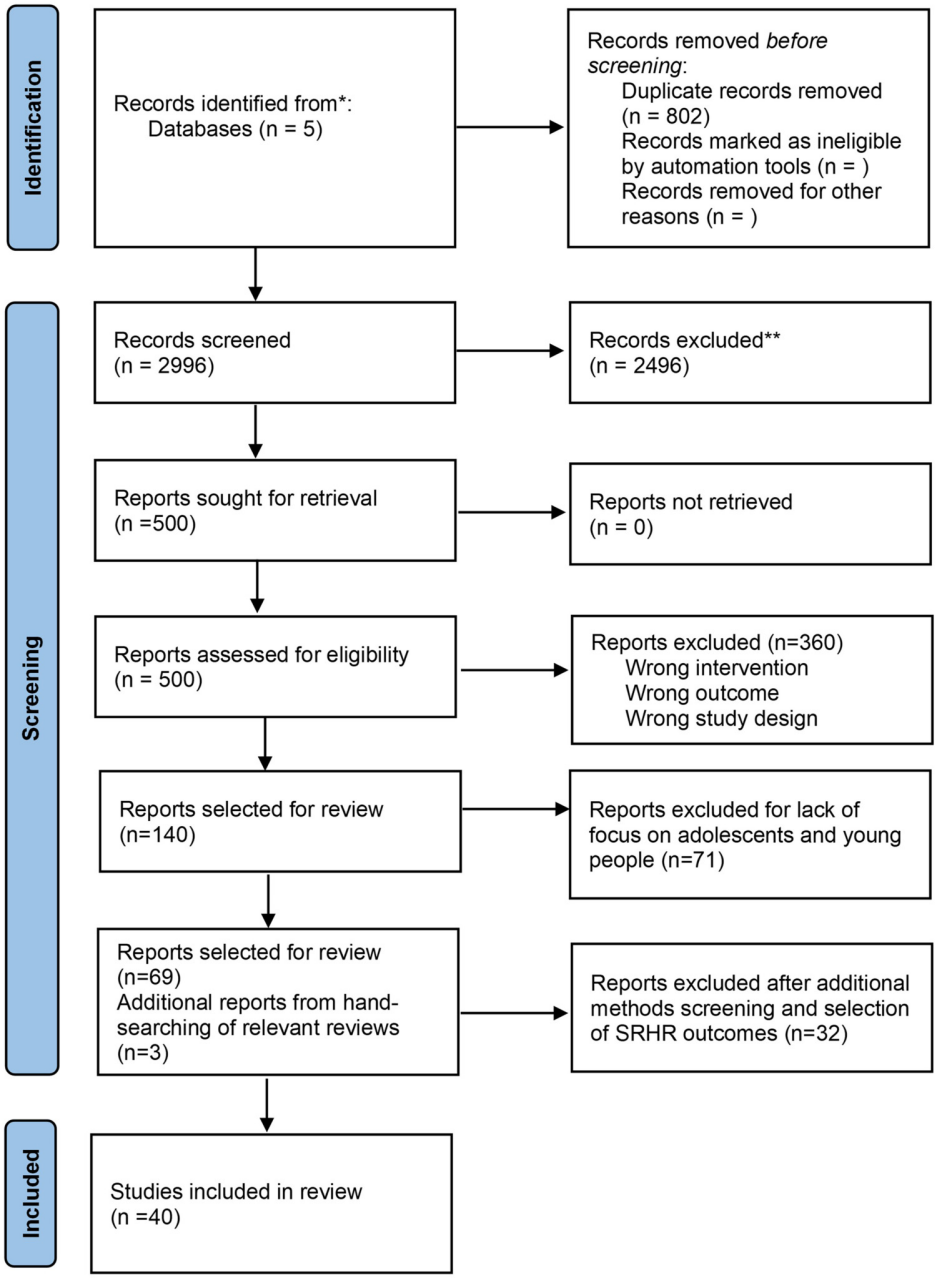
PRISMA diagram for the review.

We followed the Preferred Reporting Items for Systematic Reviews and Meta-Analyses (PRISMA) Guidelines (22). Study records were exported to Covidence, and titles and abstracts were independently screened by three researchers. Full texts were subsequently screened by the same three reviewers. Figure 1 presents the flow diagram of the study selection process. Of the 2,996 studies initially screened, our review identified 140 social norms intervention studies. We then focused on interventions specifically assessing the impacts of interventions on adolescents and young people, which included 69 studies. An additional three studies were included through hand-searching of seven systematic, scoping and narrative reviews, totalling 72 studies (23–29).

Further studies were excluded due to lack of control group for the intervention (*N* = 16 studies), ineligible study design (mediation analyses: *N* = 2; feasibility studies: *N* = 3), or for solely reporting on outcomes related to knowledge, intentions, and beliefs (*N* = 8 studies). Lastly, three studies focusing on female genital mutilation/cutting (*N* = 1), menstrual hygiene management (*N* = 1), and male circumcision (*N* = 1) were excluded due to the low amount of available evidence for each outcome, compiling a final list of 40 intervention studies. The full list of included studies is described in Supplementary Table S2.

### 2.1 Data extraction

Data extracted from the final list of studies included: paper title, author names, publication year, study country, study design, population age and gender, sample size, intervention strategy, intervention description, intervention components, combined interventions, reference groups engaged, intervention setting, SRHR outcomes of interest, effect sizes, other outcomes measured, timeline of evaluation, delivery mechanism, content, and implications for programming. Data was synthesised narratively, and statistical pooling or meta-analyses were not conducted due to the variation in study designs, interventions, and outcome measures.

**Figure 2:**
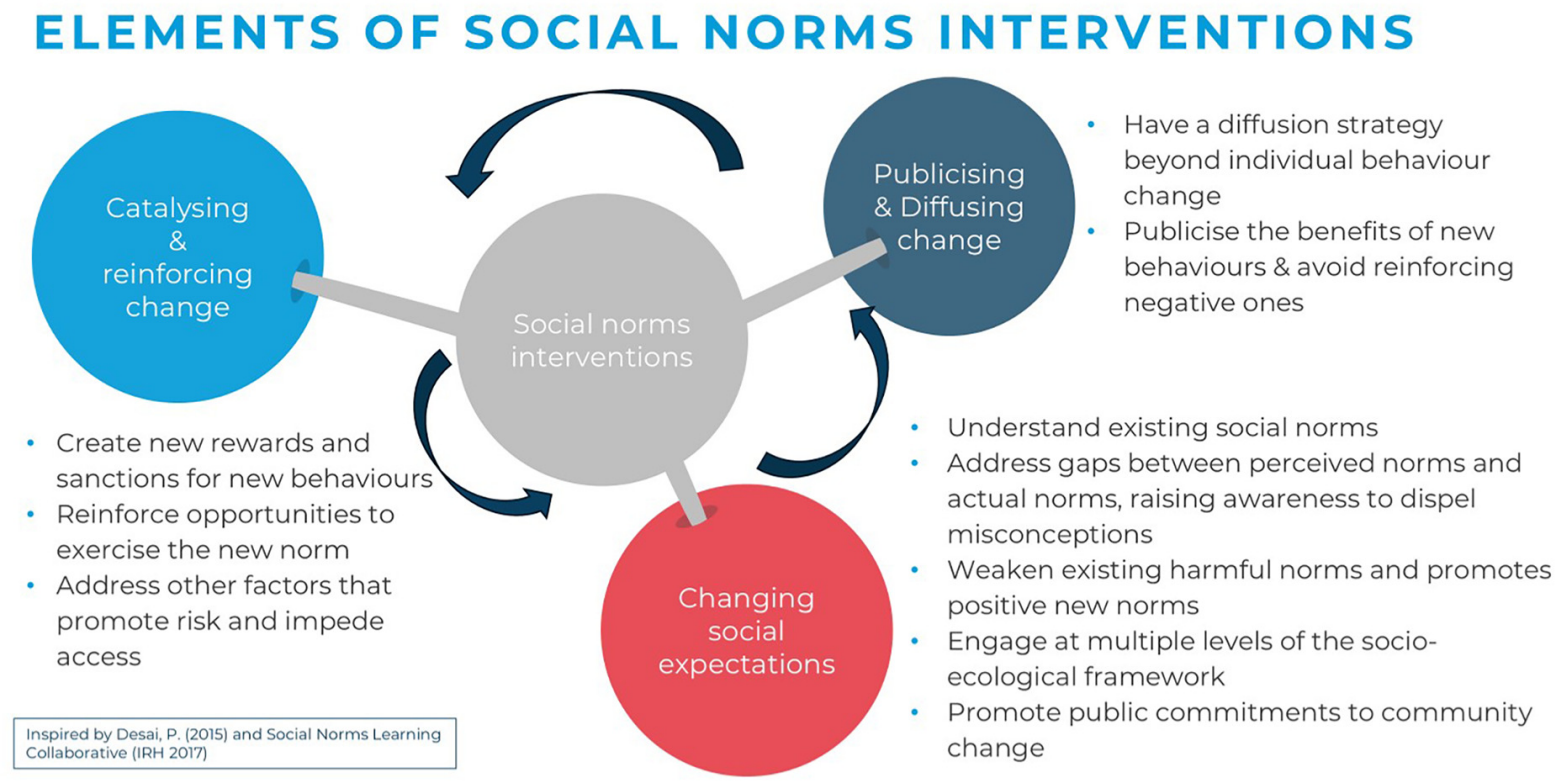
Key attributes of social norms interventions.

We categorised approaches applied in social norms interventions by strategy or entry point (e.g., life skills interventions, community dialogues, school-based programmes). To understand more about how interventions achieve norms change, we compiled a list of ten common key attributes of norms-shifting interventions, clustered around three elements informed by theory, practice, and insights from the work of the Learning Collaborative to Advance Normative Change and a three-stage framework of successful approaches to shifting harmful social norms described in a DFID 2016 Guidance Note for tackling violence against women and girls (30, 31). The three elements are: (i) changing social expectations, (ii) publicising and diffusing change, and (iii) catalysing and reinforcing change, with the full list of attributes assessed for each intervention outlined in Figure 2 below. Details on the criteria for attribute assessment are included in Supplementary Table S3.

All 40 eligible studies were reviewed to map whether each key attribute was reported. Attributes were assessed through a review of the main intervention publications, any additional programmatic information including study protocols, accompanying papers, theories of change, curricula and appendices when available online or linked to the main publication. Where the available information was not clear, partial, or missing, we marked the attribute as not reported.

## 3 Results

Among 2,996 articles identified by our search, 40 studies on 36 programmes met the review criteria. These included 25 RCTs and 15 quasi-experimental design studies or cross-sectional designs with comparison groups. The individual countries with the largest number of studies were South Africa (13), Kenya (6), and Uganda (5). Countries also included Ethiopia (3), Tanzania (3), Zambia (3), Niger (2), Nigeria (2), Zimbabwe (2), DRC (1) and Burkina Faso (1).

Six studies measured outcomes among all adolescents and young people aged 10–24, five among young adolescents 10–14 years old, 14 among adolescents 10–19 years (a few of these studies included participants younger than 10 and/or up to 20 years), and 15 studies targeted older adolescents and young people aged 14–24 (including studies with participants up to 28). Few programmes focused on adolescents and young people with additional vulnerabilities or living in vulnerable settings. Thirty-one studies engaged community members, parents and caregivers, partners, healthcare providers, religious, cultural, and community leaders, or teachers and school staff as reference groups of adolescents and young people, to create a supportive environment for the intervention and desired norms change.

**Figure 3:**
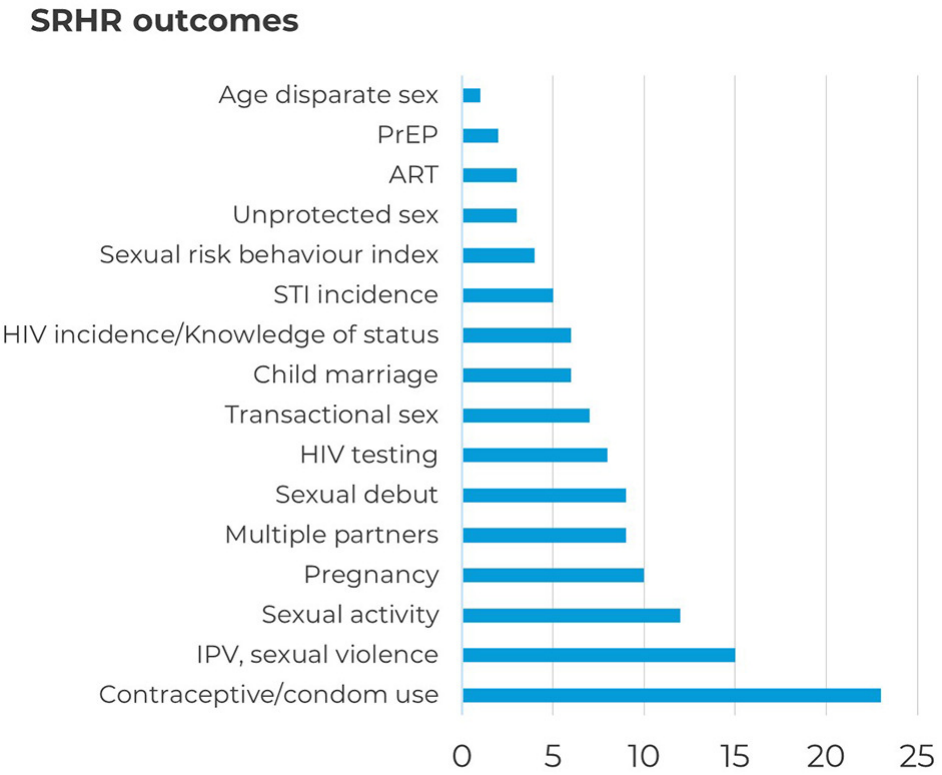
SRHR outcomes of included interventions.

Figure 3 breaks down the number of studies per outcome measure. We categorised these outcomes into six areas: violence, HIV, marriage, sexual risk behaviours, contraception, and early pregnancy. Besides SRHR outcomes, several studies also measured outcomes related to gender-equitable attitudes (*N* = 17) and knowledge about an SRHR topic (*N* = 18).

In the following sections, we present an overview of the interventions included, evaluate their effectiveness in reducing experiences of violence and improving specific SRHR outcomes, examine the evidence by intervention strategy, and highlight key insights.

### 3.1 Overview of included interventions

Interventions generally included multi-component community-based approaches that engaged adolescents and young people and their reference groups and targeted multiple SRHR outcomes. Included interventions were broadly grouped into eight categories: life skills training (23), community dialogues (14), school-based SRHR programming (10), parenting programmes (8), media or digital-based approaches (7), interventions with religious, cultural or traditional leaders (5), training of healthcare professionals to provide youth-friendly services (7), and rights-based advocacy (2). All studies included specific elements aimed at addressing social and gender norms, and most delivered a variety of other content. Twenty-five studies that met the inclusion criteria were multi-component interventions. In 15 of the programmes reviewed, norms interventions were combined with other types of interventions, which fell into two major categories: economic support (7) and healthcare service delivery (4), or both (4). Sixteen studies evaluated effects of interventions over a period of one year or less, and 10 studies evaluated outcomes after 3 years or more.

**Figure 4:**
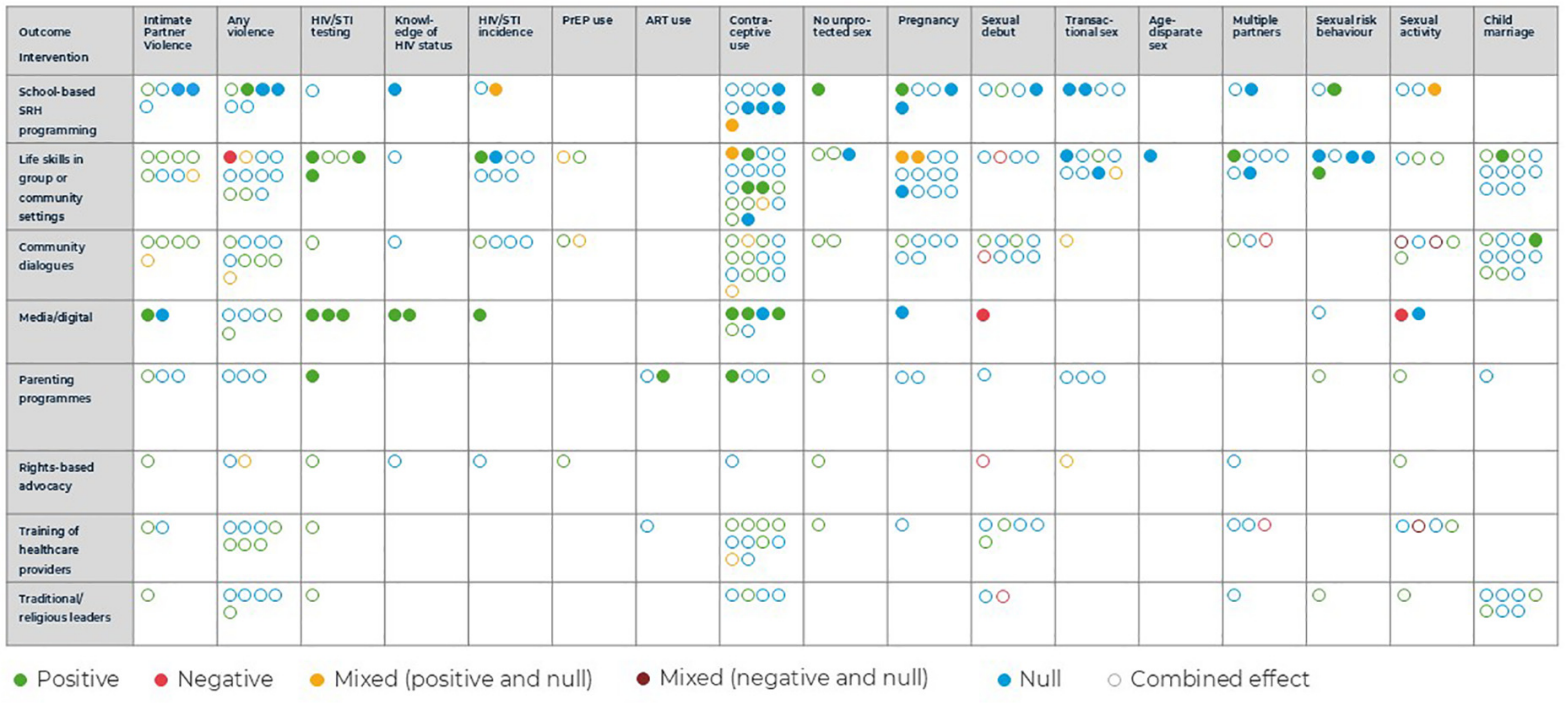
Effectiveness of included interventions.

Twenty-six studies showed significant improvements in indicators measured in at least one of the intervention arms, without any negative impact. Nine studies had null effects across outcomes, or null effects in all but one outcome when the study assessed more than 10 indicators. Five studies had negative effects in one or two outcomes, combined with a mix of other positive and null results. Figure 4 summarises the findings regarding the effectiveness of interventions by category and outcome. The direction of results is framed in terms of SRHR objectives, that is, a positive effect on GBV would represent a decrease in rates of GBV. Improvements in SRHR among adolescents and young people included (i) reductions in GBV and child marriage, (ii) the increase in access to, and use of services (for example, increased HIV testing and use of PrEP), (iii) reduced sexual behaviours that increase their risk of HIV/STI incidence (for example, transactional, age-disparate, multi-partner, and condomless sex).

Results are represented for each outcome as positive, negative, mixed, and null (green, red, yellow or dark red and blue). Effects of individual interventions were captured by solid circles, while combined interventions are represented by hollow circles. These could be related to a combined package of interventions whose components are all included in the table (multiple norms components), or to non-norms intervention components not included in the table (such as a cash transfer). Interventions that included separate intervention arms, or that assessed outcomes on different age or gender groups as their primary results (that is, did not report pooled effects across all intervention populations) were represented as different circles. Mixed effects comprise a mix of positive and null (yellow circles), or negative and null results (dark red circles), when more than one measurement per outcome category was assessed.

### 3.2 Interventions by SRHR outcome

#### 3.2.1 Violence outcomes

We identified 14 studies measuring violence outcomes. Eight studies measured reported experiences of IPV over the past month to 2 years, or using an index of frequent exposure (32–39). Two studies included measures of severe IPV, and one measured separate physical, verbal, and psychological components of IPV. Sexual violence was the second most commonly reported outcome (*N* = 6) (40–45). A majority of the studies measured any sexual abuse or rape, not specifying whether from partners or non-partners. Lastly, two studies reported measures of experiencing violence from police (study participants were sex workers) and using physical punishment against children (study participants were young fathers) (35, 39). Studies included 10 life skills interventions, seven community dialogue interventions, five parenting programmes, four school-based SRHR programmes, and three interventions training healthcare providers.

Ten interventions led to statistically significant improvements in at least one violence outcome assessed in at least one of the intervention arms, including four that found reductions across all arms and populations where effects were assessed (32–37, 39, 41, 44, 45). Three studies found no significant effects in any intervention arm, while one life skills intervention showed an increase in violence reports. However, the authors of this study described the increase in reports as indication of girls being “more likely to come forward and report sexual abuse to their mentors” (40). Community dialogues, consistently applied with other interventions, showed the most evidence of effectiveness in terms of reduction in violence across outcomes in this area. Effective interventions included *PREPARE* in South Africa, the *Responsible, Engaged, and Loving (REAL) Fathers* intervention in Uganda, and an intervention layering a football activity for males and a goal-setting activity for girls on top of the *Empowerment and Livelihood for Adolescents (ELA)* clubs in Tanzania (33, 36, 39).

Of the six studies separately measuring sexual violence outcomes, three interventions effectively reduced experiences of sexual violence (41, 44, 45). Two of these were an individually delivered school-based SRHR programme in South Africa and a combination of a school-based programme with community dialogues and healthcare provider training in Kenya. Across the studies evaluating interventions' effectiveness on IPV outcomes, community dialogues and life skills training in group-based settings showed the most evidence of effectiveness in combined approaches, including when applied together.

While most interventions measuring violence outcomes combined multiple approaches, two single-component interventions showed positive effects on violence outcomes. A digital intervention offering young women a gamified Whatsapp chatbot that engaged them in critical reflection on power and unhealthy relationship behaviours and built their self-efficacy to protect themselves, led to significant reductions in IPV (32). The school-based *Let Us Protect Our Future* intervention in South Africa also reduced reports of forced sex perpetration over several follow-ups. The content of the intervention included gender issues and rape myth beliefs, and the programme sought to increase adolescents' self-efficacy to avoid risky situations (36).

Of the 10 interventions associated with significant improvements in at least one violence outcome, only three described programmes with social norms components combined with other intervention components unrelated to social norms, of which two offered health services provision (33, 35, 36). In the included studies, the layering of economic empowerment components did not appear to be associated with higher likelihood of effectiveness.

Programme content in effective interventions was delivered through a variety of modes and often prompted reflections on gender roles, joint problem-solving and power in relationships, nonviolent responses to couple conflict and communication, and self-efficacy to avoid risky situations. For instance, the *Reaching Married Adolescents* intervention in Niger combined health and life skills training including sessions on gender norms and female autonomy, couple communication regarding fertility decisions, and GBV, with community dialogues involving key influencers to create a supportive environment for married adolescent girls and their husbands (34). The intervention effectively reduced reports of IPV across two arms of the study, with one of these arms also including home visits by community health workers.

The evidence points to the effectiveness of interventions engaging key reference groups such as parents or caregivers, partners, community members, religious leaders, and peers, including men and boys, for interventions focused on adolescent girls and young women. Of the 10 effective interventions, eight engaged with one of these reference groups, highlighting the importance of working with them (33–37, 39, 41, 45). Four interventions involved religious, traditional or cultural leaders, and three of these were effective (34, 41, 43, 45). However, their level of engagement varied greatly, from helping design interventions to general support in organising community dialogues. An effective example is the *Gender Roles, Equality and Transformation* intervention in Uganda, which implemented an iterative community mobilisation process. It engaged community leaders to reflect on gender inequality, violence, and SRHR, to identify priority issues, and to make plans with their communities. The programme, which also trained healthcare providers and included a radio-based intervention and a life skills training component in safe spaces, reduced violence among older participants but not among very young adolescents (45).

#### 3.2.2 Other SRHR outcomes

A majority of violence prevention interventions included in this review also sought to improve other SRHR outcomes. Many aimed to tackle the same underlying or foundational gender inequalities that drive GBV and other poor SRHR outcomes. Understanding how social norms interventions can address multiple outcomes can highlight the importance of investing in these interventions to foster more equitable and supportive environments for young people.

##### 3.2.2.1 Child marriage

Six studies included in the review assessed the effects of social norms interventions on whether girls were ever married or got married during the intervention period (40, 42, 46–49). Four studies found effectiveness in at least one intervention arm, while two other studies had null results across arms (40, 46–48). Although studies were concentrated within three categories of interventions (life skills, community dialogues, and interventions with leaders) and more evidence is needed to assess the effectiveness of other strategies, the existing evidence showed that community dialogues, combined with other interventions, were the most effective type of norms intervention. Effective interventions engaged community members in dialogue to reflect on the consequences of marriage and the value of girls' education and dispel common myths surrounding the practice.

Most studies evaluated combined interventions. However, the *Sista2Sista* programme demonstrates an effective example of a single-component life skills training intervention that decreased the likelihood of getting married under 18 years of age. In the girls-only clubs evaluated in the study, girls completed interactive exercises on topics including gender, power, and traditional and cultural practices, and reflected on their rights and the consequences of child marriage (40).

##### 3.2.2.2 HIV outcomes

Seventeen studies measured outcomes related to HIV and STIs, including HIV/STI testing, HIV incidence, knowledge of own HIV status, pre-exposure prophylaxis (PrEP) uptake, continuation and adherence, antiretroviral therapy (ART) adherence and viral suppression, HSV-2 infection/serostatus, and STI prevalence (35, 37, 40, 43, 50–62). Thirteen interventions led to statistically significant improvements in at least one outcome assessed in at least one of the intervention arms (35, 37, 40, 43, 50–55, 58, 60, 62). Effective intervention studies included an action research study including parenting and life skills programme arms in Nigeria, the *Ujana Salama* multi-component intervention in Tanzania, and the *VUKA* family intervention in South Africa (53, 54, 60).

Interventions proved to be highly effective for increasing HIV testing, had mixed results on knowledge of status, and obtained mostly null results in attempts to lower HIV incidence (40, 43, 50, 52–55). Life skills approaches and media or digital-based interventions including social norms components showed the most evidence of effectiveness in terms of increasing HIV testing rates, however, there is also some evidence pointing to the effectiveness of social norms interventions as part of parenting programmes, community dialogues, rights-based advocacy approaches, healthcare provider training, and interventions with religious or cultural leaders. We found very little experimental evidence on the uptake, usage, or adherence to PrEP and ART.

The effective life skills interventions were almost always described as offering safe spaces for young people, and these included both single-sex and mixed-sex youth clubs. In the context of SRHR, safe spaces offer the opportunity to discuss sensitive matters and obtain services in a safe and non-stigmatised way (63).

Media or digital-based approaches also show some positive effects on knowledge of HIV status and HIV incidence, as well as testing. Two of these studies evaluate the implementation of *MTV Shuga Down South*, a mass media educational drama series broadcast on national television in South Africa. The series covered topics related to HIV prevention, family planning, sexual identity, and safe and healthy sexual relationships in a destigmatising manner through the show's characters (50, 52).

##### 3.2.2.3 Sexual risk behaviours

Twenty studies included had an outcome measure related to sexual risk behaviours. This included thirteen studies that examined outcome measures related to sexual debut and sexual activity (33, 36, 41, 43, 49, 50, 54, 55, 58, 62, 64–66). Seven studies measured the effects of interventions on transactional sex (defined as exchanging sex for money, food, or gifts), which often increases risks of violence for girls and women (35, 38, 42, 43, 49, 51, 59). Six studies measured effects of interventions on having multiple partners (41, 43, 51, 54, 58, 59). Lastly, having age-disparate sex was an outcome in only one study and was defined as a sexual partnership with an age gap larger than 5 years (51).

Included studies showed little effectiveness of social norms interventions on sexual risk behavioural outcomes—only six out of 20 studies in this outcome area effectively reduced sexual risk behaviours without leading to negative effects in other outcomes (35, 36, 51, 56, 58, 64). The interventions showed some more promising results in terms of delaying sexual debut and reducing sexual activity, although some studies also led to the opposite effect. The evidence showed mostly null effects of interventions on having multiple sexual partners, transactional sex, and age-disparate sex (despite limited evidence on this outcome). No particular intervention strategy stands out as more effective in shifting these behaviours. Effective interventions included the *Let Us Protect Our Future* intervention in South Africa and the *Research Initiative to Support the Empowerment of Girls (RISE)* programme in Zambia (58, 64).

Four interventions had negative effects on sexual risk behaviour outcomes but reported no explanation for these negative results. These negative outcomes were sometimes used to indicate the need for design adaptations in the next iterations of the programmes (41, 43, 49, 50).

The six effective interventions in this area included four life skills training components, which were delivered in both single and mixed-sex groups. Nearly all (5/6) engaged multiple reference groups, even when they were single-component interventions (35, 36, 56, 58, 64). Regardless of the intervention category, these effective programmes engaged adolescents and young people through interactive, youth-friendly methods, including sports-based activities, brainstorming, role-playing, and games. Effective interventions focused on the impact of risky behaviours on young people's dreams and aspirations, encouraged them to set goals and consider the consequences of risky sexual behaviours, and provided knowledge and skills to protect themselves and avoid risky situations.

##### 3.2.2.4 Contraception and family planning

Twenty-three studies measured outcomes related to contraceptives and family planning, including current use of modern contraceptives or family planning methods, condom use at last or first sex, and consistent condom use (33–35, 38, 40, 41, 43, 45, 49–56, 58, 59, 64, 67–70). Included interventions showed significant effectiveness in improving sexually active young people's use of contraceptives. The evidence points to the high potential of media-based interventions, community dialogues, training of healthcare providers, and life skills programmes. Fifteen studies found effectiveness across at least one intervention arm or group of participants, while eight showed null effects (34, 35, 38, 40, 41, 45, 50–53, 55, 56, 64, 68, 69). The effectiveness of community dialogues and healthcare interventions was always tested in combination with other components, while media or digital interventions were often delivered in isolation (34, 35, 41, 50, 52, 69).

Effective interventions including community dialogues often described engaging key influencers and gatekeepers, both at the community and the individual level, from young people's perspectives. The interventions used trained facilitators to convene the dialogues, and nearly all of them used a multimedia strategy to engage participants, including radio, theatre, and video production. One effective intervention also explored the use of positive deviants by highlighting examples of key influencers who did not practice a certain risky behaviour (68).

Three media or digital-based single-component intervention studies also showed improvement in contraceptive use outcomes, including two studies *on MTV Shuga Down South*, and one study that examined a text-messaging-based programme with a primary HIV prevention target. The programme, which led to higher rates of condom-protected sex, delivered five to 10 text messages to young people daily for seven weeks, covering HIV information and behavioural skills but also addressing societal expectations for gendered sexual interactions between males and females, healthy relationships, and communication strategies. It also integrated two game-like features, encouraging behavioural outcomes, highlighting the role of interactive approaches in digital interventions (55).

##### 3.2.2.5 Early pregnancy

Ten studies measured the effects of interventions on outcomes related to pregnancy among adolescent girls and young women (38, 40, 48, 49, 51, 52, 54, 59, 62, 71). Four studies reported positive effects in at least one arm and outcome, five had null effects, and one found a mix of negative and null results (40, 48, 51, 71). Two studies provide effective examples of single-component interventions. A 6-week curriculum and school-based SRHR programme that focused on empowerment and self-efficacy training for girls, and gender equality, positive masculinity, and bystander interventions training for boys, decreased the annual incidence of school dropout due to pregnancy in intervention schools. Similarly, the *Soul Buddyz Clubs*, introduced as a social support extracurricular activity at primary schools in South Africa, decreased experiences of pregnancy but not significantly for adolescent girls (51, 71).

### 3.3 Social norms intervention attributes mapping

**Figure 5:**
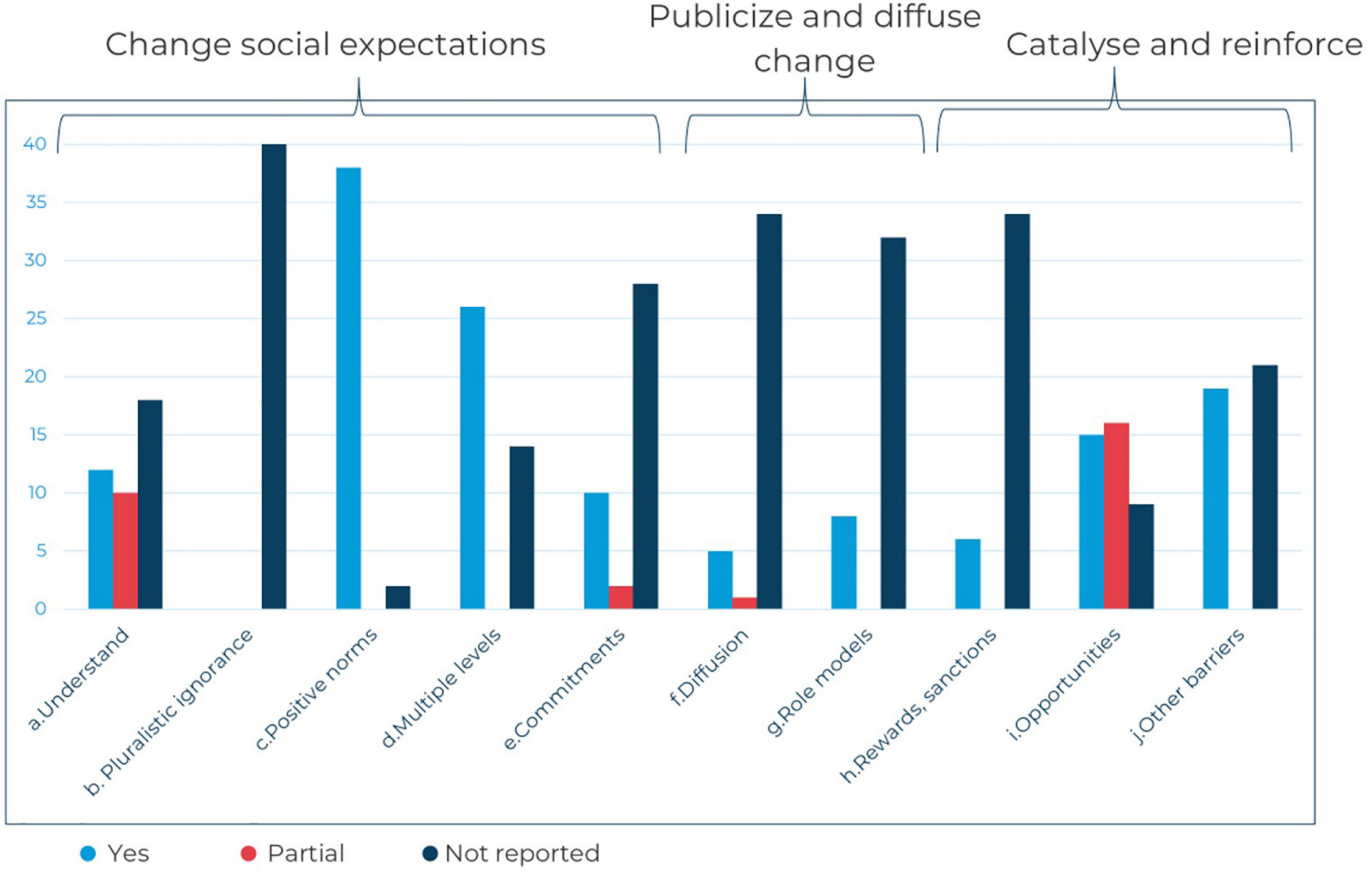
Reported attributes of social norms interventions included.

Studies included were assessed for 10 common key attributes of norms-shifting interventions, organised around three elements of change (changing social expectations, publicising and diffusing change, and catalysing and reinforcing change). These attributes describe strategies to promote positive norms and associated behaviours within intervention groups and to sustain and spread this change to a broader community. On average, the studies reported on 3.9 social norms attributes, with at most 8 attributes (see Figure 5). Fourteen out of the 40 studies reported on five or more attributes. Of these, 12 were effective in improving one or more SRHR outcomes and did not lead to any negative effects (33, 35, 37, 45–48, 53, 62, 64, 66, 68). Similarly, 14 studies reported on at least one attribute from each of the three elements, and 11 of these led to positive effects on one or more SRHR outcomes (32, 33, 37, 39, 46–48, 52, 53, 66, 68). Among effective interventions, the proportion of programmes reporting five or more social norms attributes or attributes across three elements was higher than the proportion among studies of non-effective interventions (46% vs. 14%, and 42% vs. 21%, respectively). Similar results are found in the subset of studies that include measures of violence as an outcome.

Across the 40 included studies, most interventions focused on changing social expectations and awareness, particularly by promoting positive gender norms, building agency and decision-making among young people, and encouraging healthy relationships between adolescents and young people and their reference groups.

Few interventions reported publicising and diffusing strategies to spread the expected social norms change to the wider community through media or a process of engagement of participants' networks (72). One example of a diffusion strategy includes interventions in which religious leaders were trained and mobilised to pass on messages of the interventions in their villages or places of worship (46, 66).

More often, the studies described strategies to catalyse and reinforce new norms and behaviours – 31 studies included at least one “reinforcing” attribute. Common strategies to reinforce new norms and behaviours included skills practice through role-playing and interactive group activities. Many interventions included a component aimed at improving access to healthcare services, offering participants opportunities to exercise their health-seeking behaviours and choices.

This mapping of attributes suggests that programmers should ensure that intervention designs cover all three elements to demonstrate that new proposed behaviours are, indeed, accepted and practised by a majority of the people (37, 39, 46). Specifically, comprehensive social norms change should not only promote new positive norms and behaviours but also plan for the diffusion of this change to a wider population beyond the primary target audience. Such consideration could improve the potential for longer-term transformational, sustainable norm shifts. Future research should explore further how these attributes align with effective programming for norms change.

### 3.4 Interventions by strategy

This section summarises the evidence on different intervention strategies as entry points for leveraging social norms approaches to prevent violence and poor SRHR outcomes. It describes eight strategies: life skills, school-based SRHR programming, community dialogues, media or digital-based interventions, parenting programmes, healthcare provider training, interventions with community leaders, and rights-based advocacy. For each strategy, we report on definitions and designs, evidence of impact, modes of delivery, and content.

Life skills training interventions incorporating social norms components, delivered in safe spaces or other group settings, were more likely to be effective in preventing violence and child marriage and improving HIV testing and condom use. Community dialogue interventions, in combination with other components, were also particularly effective in preventing violence, early pregnancy, and marriage, improving contraceptive use, and reducing early sexual debut and sexual activity. Mass media and digital approaches also demonstrated effectiveness, both through individual interventions and combined delivery, particularly for reducing violence, HIV prevention, and increased contraceptive use. There is emerging evidence on parenting programmes and school-based SRHR programming. Effective interventions were usually led by trained facilitators recruited from local communities, equipped with dialogue facilitation skills and gender-equitable attitudes (33, 51, 64, 69).

Across intervention strategies, common topics and curriculum content of effective interventions included reflections on gender roles and inequalities, youth sexuality, gendered power relations, girls' agency, norms underlying the acceptance of GBV, the value of girls' education, and postponement of early marriage and childbearing. This type of messaging promoting positive gender norms was often combined with practical knowledge about SRHR topics and life skills, including decision-making, conflict resolution, and goal-setting skills (36, 38, 55, 64).

Effective interventions engaged with the key reference groups influencing behaviours and SRHR outcomes of adolescents and young people. Out of the 26 studies that showed significant improvements in indicators measured across all strategies, 22 engaged some reference group beyond adolescents and young people themselves.

#### 3.4.1 Life skills training

##### 3.4.1.1 Evidence of impact

Life skills interventions including social or gender norms content were included in 23 studies. Fourteen of these interventions improved at least one SRHR outcome, with most of the effective evidence being in the areas of violence prevention, HIV testing, condom use, and marriage (34–37, 45–48, 51, 53, 62, 64, 68, 69). Six interventions found null effects across all or nearly all outcomes measured. The effects of three interventions included some negative impacts, namely an increase in reports of sexual abuse, and increases in early sexual activity among adolescents and young people (40, 43, 49). However, these interventions also found positive results in several other outcome areas, including HIV testing and contraceptive use.

##### 3.4.1.2 Definitions and designs

The group-based settings where life skills interventions were delivered were often referred to as 'safe spaces', although the definitions for this term may differ in terms of engaging boys and girls separately. In general, safe spaces can be an effective way to engage young people in reflection and discussion, offering them a place where they feel physically and emotionally protected and supported to express themselves and learn new information and skills, which can be particularly sensitive when the content is related to SRHR. By creating frequent interaction between group members, safe space interventions can also expand young people's social networks and improve their confidence to adopt and perform new and safe behaviours (73). When they engage girls and young women specifically, safe spaces are often referred to as girls' clubs, which meet regularly with a leader using various pedagogical methods to address SRHR, life skills, economic and financial outcomes, and other topics (63).

The safe space groups were often divided according to age range and sex, which aims to deliver content appropriate for participants' age and accommodate for existing norms around discussing sexuality with members of the opposite sex. The evidence suggests that these separate sex groups may be more effective in addressing social norms underpinning SRHR outcomes. Out of the 14 effective studies, 11 were implemented in separate sex group settings, with six studies convening both girls' groups and boys' groups, and the remaining five offering only girls' groups (34–37, 46–48, 53, 62, 68, 69).

##### 3.4.1.3 Modes of delivery

Effective life skills interventions were usually led by trained facilitators, who were young adults recruited from local communities. When single-sex groups were created, chosen mentors were the same sex as the safe space participants. Mentors were trained on SRHR and gender equality among other topics, and were instructed to facilitate discussions rather than teach. Three effective interventions engaged peer educators to recruit members to safe space groups (35, 47, 64).

Groups usually included 10–25 participants and met weekly over a period of 10–15 weeks in a variety of community locations, including schools and health facilities. A few effective interventions stratified group membership by age, school enrolment status, and marital or fertility status to ensure the appropriateness of content. The interventions always included interactive learning techniques, including role plays, media production, and participatory methods. Printed resources, including illustrative vignettes, toolkits, and curricula, guided participatory activities.

Life skills interventions were often delivered in combination with other components (74% of included studies with life skills components), and the evidence shows the potential effectiveness of both individual and combined delivery. They were individually evaluated in seven studies, three of which were effective in improving multiple SRHR outcomes and did not have any negative impact (36, 51, 53).

##### 3.4.1.4 Content

Common topics and curriculum content of effective life skills interventions included SRHR and life skills knowledge, rights of children and young people, gender inequalities, gender roles, sexuality, power relations, myths and misconceptions around SRHR, GBV (including how to protect oneself and how to seek help), decision-making and problem-solving skills, as well as hopes, dreams and goals.

#### 3.4.2 School-based SRHR programming

##### 3.4.2.1 Evidence of impact

Eleven interventions included school-based SRHR programming components. These school-based SRHR programmes showed some limited positive evidence on the prevention of violence, early pregnancy, and sexual risk behaviours. Six studies demonstrated positive effects in at least one outcome or intervention arm, while five studies had null results across outcomes and arms (38, 41, 44, 56, 58, 71).

##### 3.4.2.2 Definitions and designs

Among the 11 studies, six evaluated school-based interventions delivered individually, four evaluated combined interventions, and one study compared a school intervention-only arm to another arm combining it with a parenting and a life skills programme. This last study is the only one allowing for comparison between individually delivered and combined programmes. The *Skhokho for Schools* intervention was found to be individually effective in decreasing early pregnancy and increasing condom use among girls; however, when combined with workshops for caregivers and children and school-based clubs, no significant impact was found in any of the outcomes measured (38).

##### 3.4.2.3 Modes of delivery

Positive effects on at least one outcome were more likely to be seen with individual interventions. For example, the *Let Us Protect Our Future* intervention in South Africa led to a long-term reduction in forced sex perpetration, unprotected sex, and STIs. The intervention was delivered over six consecutive school days with interactive activities led by facilitators. It sought to improve HIV/STI risk reduction knowledge but also addressed gender issues and rape myth beliefs relevant to the perpetration of forced sex (44, 58).

Effective interventions were delivered by teachers (*N* = 2), university staff (*N* = 1), and facilitators from local communities (*N* = 3), all of whom received training before interventions. Intervention delivery periods ranged from six weeks to three school terms. All effective interventions were curriculum-based but always included interactive content, such as games, brainstorming, role play, skills practice, and youth-friendly workbooks.

It is important to note that school-based interventions do not reach out-of-school youth, who may be at increased risk of violence victimisation and HIV infection. To reach these populations, delivering life skills in other community-based settings may be more appropriate.

##### 3.4.2.4 Content

Effective school-based interventions often included normative content on identifying risky situations and building young people's self-efficacy in SRHR, sexuality, gender roles, gendered and intergenerational relationship power dynamics. This content was often combined with modules on correct condom use and condom-use negotiation and beliefs (41, 44, 58, 71).

#### 3.4.3 Community dialogues

##### 3.4.3.1 Evidence of impact

Interventions included community dialogues in 14 studies. They offered significant data on the combined effects of community discussions on violence prevention, contraceptive use, sexual debut, sexual activity, early pregnancy, and marriage. Community dialogues showed strong evidence of effectiveness in preventing violence and child marriage and improving contraceptive use. Across all outcomes, 12 studies showed positive effects on one or more SRHR outcomes, and no studies reported having only null effects (34, 35, 37, 39, 45–48, 62, 64, 68, 69). Two studies reported a mix of negative, positive, and null results.

##### 3.4.3.2 Definitions and designs

Effective community dialogues were frequently implemented to address gender norms, social norms around family planning among adolescent girls, gendered decision-making power and agency, gender roles and expectations, violence acceptance norms, and norms around the value of girls' education and postponement of early marriage and childbearing. These conversations aimed at prompting community members to reflect on the consequences of harmful behaviours and attitudes, dispel myths and misconceptions around SRHR, build empathy, and sensitize them to young people's SRHR challenges. The collective nature of interventions also created a way through which different groups within communities were receiving and discussing similar messages, and offered a public forum where members could commit to positive new behaviours, supporting norms change.

Five out of the 12 studies with more than one outcome improvement or with improvements across all outcomes assessed also measured intervention effects on outcomes related to norms and attitudes. Four of these found evidence of changes in attitudes, which included increased women's decision-making power, perceived community support for family planning use, and reduced odds of endorsing inequitable gender attitudes and justifying violence (39, 45, 47, 64, 68).

In all 14 studies, dialogues were tested in combination with other approaches, which often included a life skills training component. Seven of the 12 studies also incorporated a non-norms intervention component, including five providing economic support components. Erulkar and co-authors, describing the only study where community dialogues were evaluated individually, highlight some lessons in their implementation. The study compared two strategies of engaging community members in dialogue to address social norms related to child marriage and found that a structured approach using dedicated, paid facilitators and a set curriculum was more effective than a less rigorous method using existing community leaders to pass messages during routine meetings. However, the structured approach was only effective when implemented as an individual intervention, but led to null effects when combined with the payment of school fees (46).

##### 3.4.3.3 Modes of delivery

Effective community dialogues were usually led by trained facilitators recruited from local communities, trained in dialogue facilitation skills, gender equality, and SRHR content. Two effective interventions trained religious or cultural leaders to lead community conversations (45, 47). The meetings were often held in village religious or civic centres and health facilities, on a weekly to monthly basis. Community dialogue interventions were usually implemented over relatively longer intervention exposure periods of 1.5–2 years.

Some interventions described strategies for the recruitment of community members to participate and facilitators to lead sessions through formative work before intervention implementation. For example, facilitators were recommended by local community and political leaders and a local NGO partner in the *Reaching Married Adolescents* programme (69). Similarly, the *Research Initiative to Support the Empowerment of Girls (RISE)* intervention engaged cultural and religious leaders, school staff, and caregivers to support activities before recruitment and used local radio for community sensitization of the programme (64).

Nine studies on effective interventions described specifically aiming to engage “gatekeepers” or members of reference groups that exerted influence over young people's access to sexual and reproductive health (SRH) services. These included religious leaders, male partners, parents, and caregivers. Three studies described interventions that prompted community members to design action plans to address issues affecting young people (45, 49, 62).

#### 3.4.4 Parenting programmes

##### 3.4.4.1 Evidence of impact

Eight studies included parenting interventions. These studies were distributed across several outcomes, with most data on their effectiveness in reducing violence and transactional sex and increasing contraceptive use. Five interventions found improvements in SRHR outcomes, and three had exclusively null results (39, 53, 60, 64, 66). There was some evidence of improvements in HIV testing, ART adherence, contraceptive use, reduced IPV experiences, and sexual risk behaviours. Each of these, however, is supported by only one or two studies.

##### 3.4.4.2 Definitions and designs

Except for two studies, all others evaluated the effectiveness of combined interventions. The data shows similar effectiveness of these two types of implementations. Four parenting programmes were combined with life skills interventions. For instance, the *Research Initiative to Support the Empowerment of Girls (RISE*) intervention in Zambia combined parent and community meetings to improve attitudes towards the value of girls' education and postponement of early marriage and childbearing, with youth clubs offering girls and boys life skills training and an economic support component. The combined intervention arm effectively lowered sexual activity and unprotected sexual activity for girls (64).

##### 3.4.4.3 Modes of delivery

Effective interventions often engaged caregivers in group settings through established community structures such as religious congregations, as they were seen as recognised institutions attended by all family members (66). Programmes were delivered by lay counsellors and mentors following a structured curriculum. They were often theory-based and focused on discussions, problem-solving, and practising new skills through in-session rehearsal. A few studies also used multimedia aids, including posters and cartoon storylines (39, 60).

##### 3.4.4.4 Content

Parenting and family interventions covered positive communication and conflict-solving skills, alternative solutions to violence, and family goal-setting. Interventions aimed at HIV prevention or targeting participants living with HIV also included topics on youth identity, acceptance, disclosure and coping with HIV, stigma and discrimination, treatment knowledge, and caregiver-child communication on sensitive topics including HIV and puberty.

As described, only one study focused on male caregivers. The *REAL Fathers* intervention for young male caregivers with toddler children decreased perpetrations of IPV against wives, as well as psychological and verbal violence against children. The programme offered both individual and group mentoring sessions for fathers, some of which were attended by their wives, where participants engaged in self-reflection on gender roles and practised couple communication skills, joint problem-solving, and nonviolent responses. Posters capturing desired behaviours from male caregivers were also displayed in community locations (39).

#### 3.4.5 Media and digital interventions

##### 3.4.5.1 Evidence of impact

Intervention studies included seven examples of the use of media or digital-based approaches to improve adolescents and young people's SRHR outcomes. Studies were concentrated on outcome areas related to violence prevention, HIV, and contraceptive use. Four interventions found positive results, and one had null effects (32, 45, 52, 55). One study reported a mix of positive results with one negative result, namely an increase in the proportion of young people who had ever had sex (50). One social media-based intervention component was not evaluated due to low exposure (53).

##### 3.4.5.2 Definitions and designs

The evidence of the effectiveness of digital and mass media interventions is particularly strong in terms of increasing HIV testing and contraceptive use. Mass media approaches showed effectiveness in both individual and combined delivery. There was evidence of improvements in HIV and contraceptive outcomes from the single-component *MTV Shuga Down South* intervention, a television drama with storylines including information on HIV prevention options (52). Similarly, the *Gender Roles, Equality and Transformations (GREAT)* intervention increased the use of family planning and decreased violence experiences, particularly for older participants, with the combined delivery of a serial radio drama with storylines on gender, violence, and SRHR. The intervention also included a community mobilisation process, participatory activities for youth clubs, and the training of community healthcare workers (45).

One issue when evaluating mass media-based approaches is the challenge of implementing a randomised controlled trial and having a control group that does not have exposure to media messaging. Therefore, the effectiveness found in these studies should be considered carefully. At the same time, they offer insights into the use of quasi-experimental study designs to evaluate interventions without a clear control group.

##### 3.4.5.3 Modes of delivery

Interventions included four digital or social media-based approaches, such as text messaging, television, and radio-based drama interventions. Two effective interventions used text messaging-based applications and included game-like features to improve engagement. *ChattyCuz* was a WhatsApp gamified chatbot that decreased experiences of IPV for young women in South Africa. The chatbot was designed to appear approachable and build women's self-efficacy, healthy communication, and safety planning skills. Through quizzes and narratives, the intervention also offered reflections on power in relationships and was effective in improving attitudes and beliefs about power balance (32). Similarly, *InThistoGether* delivered five to 10 daily text messages to young people in Uganda for seven weeks, including content on healthy relationships and communication, and societal expectations for gendered sexual interactions. It improved rates of condom-protected sex and HIV testing. Both interventions offered symbolic rewards, such as badges for specified safe behavioural outcomes, to motivate users (55).

##### 3.4.5.4 Content

Effective media or digital-based interventions often included content on healthy relationships and prompted participants to critically reflect on power imbalance and control in relationships and societal expectations for gendered sexual interactions.

#### 3.4.6 Healthcare provider training

##### 3.4.6.1 Evidence of impact

Seven studies included a component of training healthcare professionals to provide youth-friendly services, all of which were combined with other interventions. Four studies were effective in improving a significant portion of outcomes measured (34, 45, 64, 69). Two interventions had null effects across all or nearly all outcomes evaluated. One intervention led to several positive outcomes, including decreasing rates of non-consensual sex, but also resulted in a small increase in the number of adolescents' lifetime partners (41).

##### 3.4.6.2 Definitions and designs

The lack of individually delivered healthcare training programmes hinders the evaluation of programmes' effectiveness, not allowing for the separation of the effects of these types of interventions on SRHR outcomes. Interventions' lack of focus on this type of training, which is often described as the last component of packages and with little detail on design, conflicts with adolescents' accounts that cite stigmatising attitudes and judgement by healthcare providers as reasons to avoid seeking services.

##### 3.4.6.3 Modes of delivery & content

Two of the studies on effective interventions offered no detail on the healthcare provider interventions other than the fact that healthcare teams were trained to provide youth-friendly health services. The other two studies which led to improved outcomes both described the *Reaching Married Adolescents* intervention in Niger. In this programme, community health volunteers were recruited from study villages to provide gender-matched home visits to married adolescent girls and their husbands. Prior to the visits, they received a one-week training on topics including contraceptive methods, healthy spacing of pregnancies, provision of family planning counselling, as well as gender equality, and youth and adolescent rights (34, 69).

#### 3.4.7 Interventions with community leaders

##### 3.4.7.1 Evidence of impact

Five studies included interventions with religious, cultural, or traditional leaders, all of which were delivered in combination with other components (43, 45–47, 66). Four of these studies provide evidence from violence and child marriage prevention and family planning programmes. The studies find a mix of positive and null results across all outcomes.

Two programmes engaged leaders in the intervention development with community-based participatory methods, including one that was also delivered in a religious setting following services (45, 66). Both studies show a mix of positive and null results. Two interventions targeting child marriage recruited and trained leaders as community conversation facilitators. One of these reduced child marriage rates and the other found null results (46, 47). One study had a specific focus on training religious leaders with SRH information, with the aim that they would mobilise youth involved in commercial sex to seek these services. The intervention contributed to increased HIV testing and the age of sexual debut (43).

#### 3.4.8 Rights-based advocacy

##### 3.4.8.1 Evidence of impact

Only two studies included rights-based advocacy strategies, which found a mix of positive and null results. More research on such approaches is needed to draw further evidence-based conclusions (35, 43).

## 4 Discussion

This review highlights the transformative impact of social norms interventions on improving adolescent SRHR outcomes, particularly in preventing violence and child marriage and increasing HIV testing and contraceptive use. Of the 40 included studies, 26 reported significant improvements in at least one of the intervention arms, without any negative impact. Successful interventions typically engage with a wide range of stakeholders and are often grounded in formative research and contextual understanding of social networks and local power dynamics. Our findings highlight the importance of addressing foundational social norms, particularly gender norms, to improve outcomes among adolescents and young people and how these interventions can promote multiple positive outcomes, including violence prevention. Social norms interventions have the potential to both reduce the structural drivers of harmful practices such as GBV and child marriage, as well as improve access to essential SRH services, including family planning and HIV treatment and care services. The heterogeneity of interventions reviewed, including in design, target outcomes, strategies, and combinations with other components, limits direct comparisons of their effectiveness. Nonetheless, some emerging features of successful interventions will be discussed below.

Integrating gender-transformative approaches was a key element of comprehensive programming aimed at reducing violence and improving other SRHR outcomes. The evidence suggests that many effective approaches for addressing harmful social and gender norms focused on creating or leveraging shared community spaces guided by a trained facilitator. These spaces provided a platform for discussions and critical reflection on gender inequities, gender roles, sexuality, power relations, myths and misconceptions around SRHR, and norms underpinning GBV (33, 51, 53, 68, 69).

Included studies offered insights into the most effective intervention strategies and their design. Life skills training interventions incorporating social norms components, delivered in safe spaces or other group settings, both in combination with other components or alone, were found to be effective in preventing violence and child marriage and improving HIV testing and condom use (34, 37, 47). Life skills interventions offer potential entry points to provide adolescents and young people with SRHR information, foster social support through safe spaces where experiences can be shared, and build their confidence to perform new behaviours (74). Successful approaches were found in the evaluation of single-sex life skills groups, which more easily allow for the discussion of sensitive topics including gender roles (36, 40, 68).

Community dialogue interventions, in combination with other components, were also effective in the areas of violence prevention, contraceptive use, sexual debut, sexual activity, early pregnancy, and child marriage. These interventions engaged community members in reflections on gender roles and power and built mutual understanding and shared values. By offering a setting in which different groups received and discussed the same SRHR messages, the interventions also helped dispel misconceptions about SRHR and encouraged intergenerational dialogues. The evidence showed it was often important to engage with influential reference groups within the communities, such as religious leaders, to ensure buy-in before or during intervention activities and avoid backlash (66, 69).

We found compelling data on mass media and digital approaches' effectiveness, both through individual interventions and combined delivery, particularly for reducing violence, HIV prevention, and increased contraceptive use. Through both mass and social media-based approaches, including television and radio, these interventions seek to reach a critical audience with information, be that a mass or a key targeted audience. By including content on social and gender norms, these approaches can empower adolescents and young people with information, provide role models, and create a supportive environment for norms change. This review also highlights emerging evidence on the use of digital technologies, whose potential for large-scale change, with wide reach and low cost, should be further explored (32, 75).

Our review showed some promising practice for changing social norms through parenting programmes that reduced violence and sexual risk behaviours, and improved HIV testing, ART adherence, and contraceptive use. Effective interventions often engaged adolescents and young people and their caregivers in discussions and skills-building for positive communication, non-violent conflict resolution, and reflection on harmful gender norms. A recent review of programmes aimed at reducing violence against children and IPV recommended expanding parenting programmes to involve other community members and reduce the intergenerational transmission of violence-endorsing attitudes. The review also highlighted a crucial evidence gap around studies targeting caregivers of adolescents (76).

Some promising practices were also found in examples of school-based SRHR programming, particularly in preventing sexual violence, although the overall evidence is mixed. A UNESCO report on school curricula in 10 countries in Eastern and Southern Africa found that very few interventions focused sufficiently on the influence of media on gender norms, nor addressed adolescents and young people's diversity and sexual rights. Many school-based interventions also approached sexuality in a negative way (77). Given the number of school-based interventions with null effects, more implementation research is needed to enhance their effectiveness in addressing SRHR.

Another key finding of the review was the importance of taking a socio-ecological approach and designing programmes that go beyond engagement with adolescents and young people themselves and target the reference groups influencing their behaviours and outcomes, particularly at a family and community level. Reference groups can serve as sources of correct SRHR information and create social support for young people's behaviour change. Reference groups' influence may differ according to each individual, community, or behaviour, which highlights the importance of conducting formative research including power mapping to understand the social networks that can sustain and challenge social norms.

Strategies for engaging reference groups can also help reinforce and spread changes in norms and behaviours beyond the small group of adolescents and young people directly participating in an intervention. This review highlights the need for greater focus on developing diffusion strategies to drive broader societal change. Successful approaches may include leveraging mass media or empowering participants to diffuse messages themselves within their communities. These strategies can enhance the visibility and adoption of new norms, ultimately strengthening their long-term sustainability.

Further research is needed to explore whether change is sustained past short programmatic timelines. In addition to our findings on SRHR behavioural outcomes, several of the interventions found significant improvements in measures of SRHR knowledge and intentions to adopt health-seeking behaviours. These intermediate outcomes suggest that interventions may be effective even when the short timelines are insufficient to observe actual behavioural change. This underscores the importance of designing a clear theory of change for interventions, with metrics that correspond with the duration of implementation, as well as indicators of norms change (78, 79). Longer intervention timelines may be required to achieve change in certain behavioural outcomes, allowing for longer exposure to support a change in the actual social norms underpinning behaviours and attitudes.

Although most of the studies included in this review described combined approaches, we also found a few effective single-component programmes including mass media interventions. It is also important to ensure that such interventions consider the supply-side barriers to promoting adolescents' and young people's access to services. Other non-normative supply-side factors affecting SRHR outcomes include the availability, accessibility, and quality of services. Persistent inequalities in these supply-side factors exist among adolescents and young people in sub-Saharan Africa based on education, urban-rural residence, and household economic status, underscoring the need to combine norms interventions with improved service access (3).

Whilst many social norms interventions focus on creating demand for SRH services, outcomes may not improve without also ensuring that there is an effective supply-side response, including adolescent-friendly health, education, and violence prevention and response services. Working with adult reference groups, including healthcare workers, caregivers, religious leaders, and teachers, can help address adultism^1^ norms that can be a significant barrier to accessing services and impede discussions on SRHR between adults and young people. For instance, in the delivery of healthcare provider training, interventions ensuring that the training content went beyond SRHR knowledge and included topics on gender equality and adolescent rights were shown to be effective. Further research should focus on effective strategies for training healthcare professionals to provide SRH services in a destigmatising manner that respects adolescents and young people's rights to privacy.

### 4.1 Limitations

This review has several limitations. First, whilst our review focuses on SRHR outcomes, it did not focus on the effects of interventions on SRHR-related norms, attitudes, knowledge, and awareness. As a result, potential impacts on these important outcomes were not captured. Another limitation is the inclusion of a large number of combined interventions without study designs that allowed for the separation of individual components’ contributions. This made it difficult to determine which elements of multi-faceted programs were driving observed outcomes. Additionally, due to the wide range of outcomes and intervention types, we were not able to meta-analyse studies in order to compare intervention effect sizes. Furthermore, the review was constrained by a lack of detailed information on many of the interventions' design and implementation, limiting the depth of analysis. Lastly, the exclusive focus on published experimental evidence may have restricted our ability to include certain types of interventions on which experimental evidence is still thin.

### 4.2 Evidence gaps

Several important evidence gaps emerged from this review, highlighting areas where further research is needed to better understand the effectiveness of interventions:

• Implementation research exploring how the dosage, fidelity, and quality of intervention delivery affect outcomes: Understanding how interventions are executed in real-world settings is essential to ensure their effectiveness and to guide future programmes. There is also a lack of consideration and understanding of the potential unanticipated negative consequences of interventions to address social norms in existing research. Future research should examine mechanisms to anticipate, track, and respond to potential harmful impacts of interventions and mitigate backlash that may be experienced by programme participants, early adopters of new norms, and staff members involved in programme delivery.
• Quantitative studies on certain SRHR outcomes: There is a scarcity of quantitative studies on interventions addressing outcomes such as access to medical male circumcision, ART, PrEP, menstrual health, protection from female genital mutilation/cutting and other harmful traditional practices.
• Quantitative studies on certain intervention types: Rights-based advocacy, interventions to change laws and policies, and movement-building interventions (including on women and girl-led movements) are underrepresented in the experimental literature. Discussions with policymakers including civil society networks, however, highlight the critical importance of these interventions for achieving systemic and structural changes.
• Interventions seeking to improve SRHR outcomes for marginalised adolescents and young people and key populations: Very few studies examine interventions that address the intersectionality of social norms and various dimensions of discrimination, such as race, ethnicity, disability, sexual behaviour, that affect the wellbeing of marginalised youth. There are also few studies focusing on children living in crisis and conflict-affected contexts.
• Programming and research for boys and men: While many studies focus on adolescent girls and young women, there is a notable lack of research focusing on boys and men.
• Geographical spread of evidence: Our review includes studies from 12 countries. However, most studies are concentrated in a few countries, notably South Africa, Kenya, and Uganda.
• Long-term evaluations: There is a lack of long-term evaluations to assess the sustainability of the effects of interventions over time.
• Cost-effectiveness analysis: Finally, there is a lack of data on the costs and cost-effectiveness of interventions. Understanding funding requirements and the potential for scalability of effective interventions is crucial for policymakers and funders.

Addressing these evidence gaps would provide a more comprehensive understanding of what works in interventions aiming to address social norms to improve SRHR outcomes.

## Footnotes

^1^The idea that the adult human being is in some sense superior to the child [or young person] or of greater worth, and thus the child, by default, inferior or of lesser worth. Reference: Shier H. What does ‘equality’ mean for children in relation to adults? [Official background paper for UN Global Thematic Consultation on ‘Addressing Inequalities Post-2015]. CESESMA; 2012.
