# Supplementary material for: Scoping review of social norms interventions to reduce violence and improve SRHR outcomes among adolescents and young people in sub-Saharan Africa
Source: Front Reprod Health. 2025 May 15;7:1592696. doi: 10.3389/frph.2025.1592696 (PMC12119623; doi:10.3389/frph.2025.1592696)
Supplement: Supplementary file 1 [file Table1.docx]

**Supplementary Table 1:** Description of search terms

| **Search terms** |
| --- |
| 1. Adolescent or adolescents or teen or teens or “young adults” or youth or girls or boys or “young people” or “young women” or “young men” |
| 1. Norms or “social norms” or “gender norms” or “peer norms” or “gender ideologies” or “gender attitudes” or “beliefs” or “family roles” or “family dynamics” or “power dynamics” or “positive deviants” or “perception of the child” or “control” or “patriarchy” or “adultism” or “heteronormativity” or “social hierarchy” or “gender division of labo*” or “privacy norms” or “protection norms” or stigma or discrimination |
| 1. “girls’ clubs” or “boys’ clubs” or “peer clubs” or “social marketing” or “social networks” or “sports for empowerment” or “positive masculinities” or “feminist movement” or “social movement” or “youth leadership” or “family program*” or “parenting program*” or “media campaign” or “community conversation*” or “religious leader*” or “community leader*” or “community mobili*” or “attitudes of healthcare provider*” or “regulatory change” or “multiple interventions” or “intervention” |
| 1. PLHIV or HIV or "HIV-affected" or "HIV infected" or “HIV incidence” or “sexually transmitted diseases” or STI or HPV or “sexual risk behavio*” or “early marriage” or “forced marriage” or “child marriage” or “female genital mutilation” or FGM or “female genital cutting” or FGC or “breast ironing” or “harmful gender practices” or “early pregnancy” or “adolescent pregnancy” or “unintended pregnancy” or “early sexual debut” or “contraceptive uptake” or “family planning” or “transactional sex” or “condom use” or abortion or “gender violence” or GBV or “intimate partner violence” or IPV or “violence perpetration” or “alcohol use” or “drug use” or “adolescent sensitive health workers” or “adolescent friendly health services” or “health service” or “sexual and reproductive health” or SRHR |
| 1. Africa or “Sub-Saharan Africa” or SSA or LMIC* or “Low income countr*” or “Eastern and Southern Africa” or ESAR or Burundi or Comoros or Djibouti or Ethiopia or Eritrea or Kenya or Madagascar or Malawi or Mauritius or Mozambique or Réunion or Rwanda or Seychelles or Somalia or Somaliland or Tanzania or Uganda or Zambia or Zimbabwe or Botswana or Lesotho or Namibia or Swaziland or Eswatini or “South Africa” |
