# Supplementary material for: Scoping review of social norms interventions to reduce violence and improve SRHR outcomes among adolescents and young people in sub-Saharan Africa
Source: Front Reprod Health. 2025 May 15;7:1592696. doi: 10.3389/frph.2025.1592696 (PMC12119623; doi:10.3389/frph.2025.1592696)
Supplement: Supplementary file 2 [file Table2.docx]

**Supplementary Table 2:** List of included studies

|  |  |  |  |  |  |  |  |
| --- | --- | --- | --- | --- | --- | --- | --- |
| **Authors** | **Title** | **Year** | **Country** | **Population** | **SRHR outcome** | **Norms intervention type** | **Intervention name** |
| De Filippo, A., Bellatin, P., Tietz, N., Grant, E., Whitefield, A., Nkopane, P., Devereux, C., Crawford, K., Vermeulen, B., & Hatcher, A. M | Effects of digital chatbot on gender attitudes and exposure to intimate partner violence among young women in South Africa | 2023 | South Africa | Young women (18–24 years old) | **Violence**: Past month IPV | Digital-based | ChattyCuz |
| Mathews, C., Eggers, S. M., Townsend, L., Aarø, L. E., de Vries, P. J., Mason-Jones, A. J., De Koker, P., McClinton Appollis, T., Mtshizana, Y., Koech, J., Wubs, A., & De Vries, H. | Effects of PREPARE, a Multi-component, School-Based HIV and Intimate Partner Violence (IPV) Prevention Programme on Adolescent Sexual Risk Behaviour and IPV: Cluster Randomised Controlled Trial | 2016 | South Africa | Adolescents in Grade 8 (average age 13) in public high schools | **Violence**: IPV victimisation or perpetration in last 6 months  **Sexual risk behaviours:** sexual debut; number of sexual partners in the past 6 months  **Contraception**: self-reported condom use at last sex; using contraception (other than condoms) | Multiple interventions; School-based SRH programming | PREPARE |
| Silverman, J. G., Brooks, M. I., Aliou, S., Johns, N. E., Challa, S., Nouhou, A. M., Tomar, S., Baker, H., Boyce, S. C., McDougal, L., DeLong, S., & Raj, A. | Effects of the reaching married adolescents program on modern contraceptive use and intimate partner violence: results of a cluster randomized controlled trial among married adolescent girls and their husbands in Dosso, Niger. | 2023 | Niger | Married adolescent girls (ages 13–19) and their husbands | **Violence**: past year IPV  **Contraception**: current modern contraceptive use | Multiple interventions; Community dialogues; Life skills; Training of healthcare providers | Reaching Married Adolescents |
| Oberth, G., Chinhengo, T., Katsande, T., Mhonde, R., Hanisch, D., Kasere, P., Chihumela, B., & Madzima, B. | Effectiveness of the Sista2Sista programme in improving HIV and other sexual and reproductive health outcomes among vulnerable adolescent girls and young women in Zimbabwe. | 2021 | Zimbabwe | Adolescent girls and young women 10-24 | **Violence**: reported sexual abuse  **HIV/STI**: HIV testing  **Child marriage**: likelihood of getting married; getting married under 18  **Contraception**: use of a modern family planning method  **Early pregnancy**: likelihood of falling pregnant; falling pregnant as a teenager 10-19 | Life skills | Sista2Sista |
| Chabata, S. T., Hensen, B., Chiyaka, T., Mushati, P., Musemburi, S., Dirawo, J., Busza, J., Floyd, S., Birdthistle, I., Hargreaves, J. R., & Cowan, F. M. | The impact of the DREAMS partnership on HIV incidence among young women who sell sex in two Zimbabwean cities: results of a non-randomised study. | 2021 | Zimbabwe | Young women who sell sex ages 18-24 | **Violence**: experience of violence from partners in past 12 months; experience of violence from police in past 12 months  **HIV/STI**: HIV infection; knowledge of HIV status; ever taken PrEP  **Contraception**: condom-less sex with regular partner in the past month, condom-less sex with client in the past month,  **Sexual risk behaviours:** whether selling sex was the primary means by which women support themselves; ever unable to decline sex in past month; number of sex work clients in past month | Multiple Interventions; Community dialogues, Life skills; Rights-based advocacy | DREAMS and Sisters with a Voice |
| Njue, C., Voeten, H. A. C. M., Ohuma, E., Looman, C., Habbema, D. F., & Askew, I. | Findings of an evaluation of community and school-based reproductive health and HIV prevention programs in Kenya | 2015 | Kenya | Adolescents aged 10-19 years | **Violence**: ever had non-consensual sex  **Sexual risk behaviours:** ever had sex; age at first sex; had sex in past 6 months; mean number of lifetime partners  **Contraception**: currently uses modern contraceptive; used condom at last sex; used modern contraceptive or condom at first sex | Multiple interventions; Community dialogues; School-based SRH programming; Training of healthcare providers | Kenya Adolescent Reproductive Health |
| Shah, M., Seager, J., Montalvao, J., & Goldstein, M. | Two Sides of Gender: Sex, Power, and Adolescence | 2022 | Tanzania | Females aged 10-24 and their boyfriends | **Violence**: IPV often index; IPV within last 2 years index  **Sexual risk behaviours**: sexual activity index (ever had sex, currently has partner, had partner past 2 years, total sex partners ever, hours with boyfriend in past day) | Multiple interventions; Life skills |  |
| Stark, L., Asghar, K., Seff, I., Falb, K., Puffer, E. S., Iram, D., & Annan, J. | Preventing violence against refugee adolescent girls: findings from a cluster randomised controlled trial in Ethiopia | 2018 | Ethiopia | Girls aged 13–19 years residing in refugee camps | **Violence**: exposure to sexual violence in the previous 12 months; 12-month exposure to forced sex (having sex unwillingly); unwanted sexual touching and coerced sex in the previous 12 months; 12-month exposure to physical violence; 12-month exposure to emotional violence  **Sexual risk behaviours**: engagement in transactional sex **Child marriage**: child marriage | Multiple interventions; Life skills; Parenting programme | COMPASS |
| Roberts, S. T., Hartmann, M., Minnis, A. M., Otticha, S. O., Browne, E. N., Montgomery, E. T., & Agot, K. | Breaking down relationship barriers to increase PrEP uptake and adherence among adolescent girls and young women in Kenya: safety and preliminary effectiveness results from a pilot cluster-randomized trial | 2023 | Kenya | Adolescent girls and young women 17-24 | **Violence**: Physical, sexual and emotional IPV episodes; severe IPV events, resulting in physical injury  **HIV/STI**: PrEP uptake (initiating during study period); PrEP continuation (continuing to take PrEP at study exit); PrEP adherence (number of days with a Wisepill opening during the self-reported period of PrEP use) | Multiple interventions; Life skills; Community dialogues | Tu’Washindi and DREAMS |
| Jemmott, J. B. 3rd, O'Leary, A., Jemmott, L. S., Ngwane, Z. P., Teitelman, A., Makiwane, M. B., Bellamy, S. L. | Effect of a Behavioral Intervention on Perpetrating and Experiencing Forced Sex Among South African Adolescents A Secondary Analysis of a Cluster Randomized Trial | 2018 | South Africa | adolescents aged 9 to 18 years | **Violence**: perpetrating and experiencing forced vaginal intercourse | School-based SRH programming | Let Us Protect Our Future |
| Dagadu, N. A., Barker, K. M., Okello, S. B. T., Kerner, B., & Nabembezi, D. | Fostering gender equality and reproductive and sexual health among adolescents: results from a quasi-experimental study in Northern Uganda | 2022 | Uganda | Male and female unmarried adolescents (10-14 years, 15-19 years), and married adolescents (15-19 years) and adults (over the age of 19 years) | **Violence**: touching (for boys) or having been touched (for girls) on the buttocks or breasts without permission in the past 3 months  **Contraception**: current family planning use | Multiple interventions; Media-based; Intervention with traditional/religious leaders; Community dialogues; Life skills; Training of healthcare providers | Gender Roles, Equality and Transformations (GREAT) |
| Jewkes, R., Gevers, A., Chirwa, E., Mahlangu, P., Shamu, S., Shai, N., & et al. | RCT evaluation of Skhokho: A holistic school intervention to prevent gender-based violence among South African Grade 8s. | 2019 | South Africa | Grade 8 learners aged 12-19 years | **Violence**: incidence of any IPV; severe IPV; non-partner rape  **Contraception**: condom use; contraceptive use  **Sexual risk behaviours:** transactional sex  **Early pregnancy**: ever having been pregnant | Multiple Interventions; School-based SRH programming; Parenting programme; Life skills | Skhokho |
| Ashburn, K., Kerner, B., Ojamuge, D., & Lundgren, R. | Evaluation of the Responsible, Engaged, and Loving (REAL) Fathers Initiative on physical child punishment and intimate partner violence in northern Uganda. | 2017 | Uganda | Young fathers aged 16 to 25 who have toddler-aged children (1–3 years) | **Violence**: IPV in the past 3 months (have you shouted or yelled at your wife, slapped your wife, and pushed or shoved your wife?); perpetration of psychological IPV; perpetration of verbal IPV; physical punishment against child in the past month (have you done the following to discipline the child shook him/her, shouted, yelled, or screamed at him/her, and spanked, hit, or slapped him/her on the bottom with bare hand) | Multiple interventions; Parenting programme; Community dialogue | Responsible, Engaged, and Loving (REAL) Fathers Initiative |
| Renzaho, A. M. N., Kamara, J. K., Doh, D., Bukuluki, P., Mahumud, R. A., & Galukande, M. | Do Community-based Livelihood Interventions Affect Sexual and Reproductive Health and Rights of Young People in Slum Areas of Uganda: a Difference-in-difference with Kernel Propensity Score Matching Analysis | 2022 | Uganda | Young people living in the slum areas aged 13-24 years | **Violence**: consensual sex at sexual debut; persuaded with money/gifts, tricked/ deceived, raped/forced at sexual debut; had sex in the last 12 months; no. of people had sex with in the last 12 months; consensual sex in the last 12 months; persuaded with money/ gifts, tricked/deceived, forced/ raped in sex in last 12 months  **Sexual risk behaviours:** ever had sex; age at first sex  **Contraception**: used condom at sexual debut  **HIV/STI**: ever had HIV test; got tested and knows result | Multiple interventions; Life skills; Rights-based advocacy; Community dialogues; Interventions with religious/cultural leaders | UPLIFT |
| Mantell, J. E., Smit, J. A., Exner, T. M., Mabude, Z., Hoffman, S., Beksinska, M., Kelvin, E. A., Ngoloyi, C., Leu, C.-S., & Stein, Z. A. | Promoting Female Condom Use Among Female University Students in KwaZulu-Natal, South Africa: Results of a Randomized Behavioral Trial | 2015 | South Africa | Female university students aged 18-28 | **Contraception**: number of female condoms used; proportion of female condom-protected vaginal intercourse occasions across all partners; male condom/female condom use at last sex occasion; number of vaginal intercourse occasions unprotected by male or female condoms; proportion of vaginal intercourse occasions protected by either a female or male condom | Life skills |  |
| Gage, A. J., Akilimali, P. Z., Wood, F. E., Gay, R., Padis, C. O., & Bertrand, J. T. | Evaluation of the effect of the Momentum project on family planning outcomes among first-time mothers aged 15-24 years in Kinshasa, DRC | 2023 | DRC | Nulliparous women aged 15-24 years, 6 months pregnant at baseline, and their male partners | **Contraception**: modern contraceptive use within 12 months of delivery; obtaining a contraceptive method within 6 weeks of delivery | Multiple Interventions; Life skills; Community dialogues | Momentum |
| Birdthistle, I., Mulwa, S., Sarrassat, S., Baker, V., Khanyile, D., O’Donnell, D., Cawood, C., & Cousens, S. | Effects of a multimedia campaign on HIV self-testing and PrEP outcomes among young people in South Africa: a mixed-methods impact evaluation of 'MTV Shuga Down South'. | 2022 | South Africa | Males and females 15-24 | **HIV/STI:** knowledge of HIV status (tested for HIV in the past year and received the result, or ever tested HIV positive); ever or past-year use of HIV self-test  **Sexual risk behaviours**: have had sex, ever and in the past 12 months Contraception: condom use at last sex | Mass media | MTV Shuga Down South |
| Johnson, S., Magni, S., Dube, Z., & Goldstein, S | Extracurricular School-Based Social Change Communication Program Associated with Reduced HIV Infection Among Young Women in South Africa | 2018 | South Africa | Women aged 18–28 years who ever had sex | **HIV/STI**: HIV status  **Sexual risk behaviours:** having multiple sexual partners in the past year  **Contraception**: used a condom at first sex; condom use at last sex; transactional sex; age-disparate sex  **Early pregnancy**: ever been pregnant; teenage pregnancy | Life skills | Soul Buddyz |
| Erhardt-Ohren, B., Brooks, M., Aliou, S., Osseni, A. A., Oumarou, A., Challa, S., Tomar, S., & Silverman, J. | Sustained impact of community-based interventions on contraceptive use among married adolescent girls in rural Niger: Results from a cluster randomized controlled trial. | 2023 | Niger | Married adolescent girls 13-19 years old and their husbands | **Contraception**: current use of any modern contraceptive; current use of any LARC method | Multiple interventions; Community dialogues; Life skills; Training of healthcare providers | Reaching Married Adolescents |
| Arije, O., Udoh, E., Ijadunola, K., Afolabi, O., Aransiola, J., Omoregie, G., Tomori-Adeleye, O., Ukeme-Edet, O., Fajemisin, O., Titus, R., & Onayade, A. | Combination prevention package of interventions for reducing vulnerability to HIV among adolescent girls and young women in Nigeria: An action research | 2023 | Nigeria | Females aged 15 to 24 years old | **HIV/STI**: uptake of HIV testing; STI treatment  **Contraception**: getting condoms; using family planning services | Parenting programme; Life skills; Media-based |  |
| Akande, O. W., Muzigaba, M., Igumbor, E. U., Elimian, K., Bolarinwa, O. A., Musa, O. I., & Akande, T. M. | The effectiveness of an m-Health intervention on the sexual and reproductive health of in-school adolescents: a cluster randomized controlled trial in Nigeria. | 2024 | Nigeria | In-school adolescents aged 10–19 years | **Sexual risk behaviours**: practice of risky sexual behaviour (defined as reporting one or more of the following: multiple sexual partners, exchange of material gift or money for sex, inconsistent/incorrect/non-use use of condoms at least once during sexual intercourse, getting infected by an STI, and sexual debut before the age of 18 years) | Digital-based, School-based SRH programming | Family Life and HIV Education |
| Sarnquist, C., Sinclair, J., Mboya, B. O., Langat, N., Paiva, L., Halpern-Felsher, B., Golden, N. H., Maldonado, Y. A., & Baiocchi, M. T. | Evidence That Classroom-Based Behavioral Interventions Reduce Pregnancy-Related School Dropout Among Nairobi Adolescents | 2017 | Kenya | Girls aged 13 to 20 years | **Early pregnancy**: school dropouts due to pregnancy | School-based SRH programming |  |
| Austrian, K., Soler-Hampejsek, E., Kangwana, B., Maddox, N., Diaw, M., Wado, Y. D., Abuya, B., Muluve, E., Mbushi, F., Mohammed, H., Aden, A., & Maluccio, J. A. | Impacts of Multisectoral Cash Plus Programs on Marriage and Fertility After 4 Years in Pastoralist Kenya: A Randomized Trial. | 2022 | Kenya | Girls aged 11-14 years | **Child marriage**: ever been married  **Early pregnancy**: ever been pregnant; ever given birth | Multiple interventions; Community dialogues; Life skills | Adolescent Girls Initiative-Kenya (AGI-K) |
| Waidler, J., Gilbert, U., Mulokozi, A., & Palermo, T. | A "Plus" Model for Safe Transitions to Adulthood: Impacts of an Integrated Intervention Layered onto A National Social Protection Program on Sexual Behavior and Health Seeking among Tanzania's Youth. | 2022 | Tanzania | Adolescents aged 14–19 years | **Child marriage**: ever married  Sexual risk behaviours: ever had sex; age of sexual debut; number of sexual partners in last 12 months; had concurrent sexual relationships in the last 12 months  **Early pregnancy**: current pregnancy status (female); ever pregnant (females); ever gotten a female pregnant (males)  **Contraception**: used condom at last sex; currently using contraceptive; currently using modern contraceptive  **HIV/STI:** tested for HIV in last 12 months | Multiple interventions; Life skills; Training of healthcare providers | Ujana Salama |
| Harrison, A., Hoffman, S., Mantell, J. E., Smit, J. A., Leu, C. S., Exner, T. M., & Stein, Z. A | Gender-focused HIV and pregnancy prevention for school-going adolescents: The Mpondombili pilot intervention in KwaZulu-Natal, South Africa | 2016 | South Africa | Boys and girls in grades 8–10 (ages 14–17) | **Contraception**: Condom use at last sex | School-based SRH programming | Mpondombili |
| Ybarra, M. L., Agaba, E., & Nyemara, N. | A Pilot RCT Evaluating InThistoGether, an mHealth HIV Prevention Program for Ugandan Youth | 2021 | Uganda | 18–22-year-old youth | **Sexual risk behaviours:** how many times had vaginal/anal sex in past 3 months  **Contraception**: times having condom-protected sex  **HIV/STI:** HIV testing in the past 3 months | Digital-based | InThistoGether (ITG) |
| Sidamo, B., Negussie, N., Hussen, S., Shimbre, M. S., Zerihun, E., Boynito, W. G., Abebe, S., Shibiru, T., Shibiru, S., Gebretsadik, W., Desalegn, N., Oumer, B., Temesgen Birgoda, G., & Abdulkadir, H. | Effectiveness of curriculum-based sexual and reproductive health education on healthy sexual behaviors among year one students at Arba Minch University: A quasi-experimental study | 2023 | Ethiopia | Year one undergraduate students 19-24 years | **Sexual risk behaviours:** sexual risk behaviour scale (Have you ever had sexual intercourse without a condom? During your life, with how many people have you had sexual intercourse? During the past 3 months, with how many people did you have sexual intercourse? The last time you had sexual intercourse, did you or your partner use a condom? The last time you had sexual intercourse, what one method did you or your partner use to prevent pregnancy?)  **HIV/STI**: ever screen for HIV  Contraception: consistent and correct use of condoms, contraception utilization | School-based SRH programming | Curriculum-based sexual and reproductive health education (CBSRHE) |
| Denison, J. A., Packer, C., Nyambe, N., Hershow, R. B., Caldas, S., Miti, S., Sudarsan, S., Chen, M., Bernholc, A., Mwansa, J. K., & McCarraher, D. R. | Family Connections randomized controlled trial: assessing the feasibility and acceptability of an intervention with adolescents living with HIV and their caregivers in Ndola, Zambia | 2022 | Zambia | Adolescents 15-19 years and on ART > 6 months and their caregivers | **HIV/STI:** ART adherence, viral failure | Training of healthcare providers; Parenting programme | Family Connections |
| Puffer, E. S., Green, E. P., Sikkema, K. J., Broverman, S. A., Ogwang-Odhiambo, R. A., & Pian, J. | A Church-Based Intervention for Families to Promote Mental Health and Prevent HIV Among Adolescents in Rural Kenya: Results of a Randomized Trial | 2016 | Kenya | Adolescents ages 10 to 16 and caregivers | **Sexual risk behaviours:** having ever had vaginal intercourse; having had high risk sex in the past 3 months (defined by not using a condom during at least one sexual encounter and/or having more than one sexual partner in that time period) | Parenting programme; Intervention with traditional/religious leaders | READY |
| Jemmott, J. B., 3rd, Jemmott, L. S., O'Leary, A., Ngwane, Z., Lewis, D. A., Bellamy, S. L., Icard, L. D., Carty, C., Heeren, G. A., Tyler, J. C., Makiwane, M. B., & Teitelman, A. | HIV/STI Risk-Reduction Intervention Efficacy With South African Adolescents Over 54 Months | 2015 | South Africa | Grade 6 learners | **Sexual risk behaviours**: having unprotected vaginal intercourse in the past 3 months; vaginal sex; multiple partners; heterosexual anal sex  **Contraception**: consistent condom use; frequency of condom use; condom use at last sex  **HIV/STI:** biologically confirmed curable STIs; HSV-2 serostatus | School-based SRH programming | Let Us Protect Our Future intervention |
| Burke, H. M., Chen, M., Murray, K., Bezuidenhout, C., Ngwepe, P., Bernholc, A., & Medina-Marino, A. | The effects of the integration of an economic strengthening and HIV prevention education programme on the prevalence of sexually transmitted infections and savings behaviours among adolescents: a full-factorial randomised controlled trial in South Africa | 2020 | South Africa | Adolescents 14-17 years | **HIV/STI:** STI prevalence (positive test result for gonorrhoea, trichomoniasis or chlamydia infection)  **Sexual risk behaviours**: engaging in protective sexual behaviour (abstinence or a condom used every time over the past 6 months); engaging in transactional sex in the past 6 months; having two or more sexual partners in the past 6 months  **Early pregnancy**: pregnancy | Multiple interventions; Life skills | Vhutshilo 2.0 and Impumelelo |
| Kemigisha, E., Bruce, K., Ivanova, O., Leye, E., Coene, G., Ruzaaza, G., Ninsiima, A., Mlahagwa, W., Nyakato, V., & Michielsen, K. | Evaluation of a school based comprehensive sexuality education program among very young adolescents in rural Uganda | 2019 | Uganda | Very young adolescents aged 10-14 years | **Sexual risk behaviours**: ever had sex | School-based SRH programming |  |
| Bhana, A., Mellins, C. A., Petersen, I., Alicea, S., Myeza, N., Holst, H., Abrams, E., John, S., Chhagan, M., Nestadt, D. F., Leu, C.-S., & McKay, M. | The VUKA family program: Piloting a family-based psychosocial intervention to promote health and mental health among HIV infected early adolescents in South Africa | 2014 | South Africa | Very young adolescents aged 10-13 years | **HIV/STI**: youth adherence to ART (how often missed medications over past 6 months) | Parenting programme | VUKA family intervention |
| Hegdahl, H. K., Musonda, P., Svanemyr, J., Zulu, J. M., Grønvik, T., Jacobs, C., & Fos, I | Effects of economic support, comprehensive sexuality education and community dialogue on sexual behaviour: Findings from a cluster-RCT among adolescent girls in rural Zambia | 2022 | Zambia | Girls in grade 7 | **Sexual risk behaviours**: sexual activity in the previous four weeks; unprotected sexual activity in the previous four weeks  **Contraception**: recent use of modern contraceptives among those reporting ever having been sexually active; current use of modern contraceptives among those who were sexually active in the previous four weeks | Multiple Interventions; Community dialogues; Parenting/couples programme; Life skills in safe spaces; Training of healthcare providers | Research Initiative to Support the Empowerment of Girls (RISE) |
| Pike, C., Coakley, C., Ahmed, N., Lee, D., Little, F., Padian, N., & Bekker, L. G. | Goals for girls: a cluster-randomized trial to investigate a school-based sexual health programme amongst female learners in South Africa | 2023 | South Africa | Female adolescents non-pregnant in secondary school grades 8–10, aged 8-21 years | **HIV/STI:** STI prevalence; HIV status  **Early pregnancy:** pregnancy  **Contraception**: contraceptive use | Life skills; School-based SRH programming | SKILLZ Streets |
| Austrian, K., Soler-Hampejsek, E., Behrman, J. R., Digitale, J., Hachonda, N. J., Bweupe, M., & Hewett, P. C. | The impact of the Adolescent Girls Empowerment Program (AGEP) on short and long term social, economic, education and fertility outcomes: a cluster randomized controlled trial in Zambia | 2020 | Zambia | Never-married girls aged 10-19 | **Contraception**: used condom at first sex  **Sexual risk behaviours:** having had transactional sex (evaluated among girls aged 15 years and older who had initiated sex); ever had sex  **Early pregnancy**: ever been pregnant; ever given birth  **Child marriage:** ever been married (evaluated among girls aged 15 years and older) | Multiple interventions; Life skills | Adolescent Girls Empowerment Program (AGEP) |
| Kangwana, B., Austrian, K., Soler-Hampejsek, E., Maddox, N., Sapire, R. J., Dibaba Wado, Y., Abuya, B., Muluve, E., Mbushi, F., Koech, J., & Maluccio, J. A. | Impacts of multisectoral cash plus programs after four years in an urban informal settlement: Adolescent Girls Initiative-Kenya (AGI-K) randomized trial | 2022 | Kenya | girls 11–14 years | **Sexual risk behaviours**: ever had sex  **Early pregnancy**: ever been pregnant or given birth;  **HIV/STI:** tested positive for HSV-2 at endline; tested positive for HSV-2 at endline having tested negative at baseline  **Contraception**: contraceptive use | Multiple interventions; Community dialogues; Life skills | Adolescent Girls Initiative-Kenya (AGI-K) |
| Erulkar, A., Medhin, G., Weissman, E., Kabore, G., & Ouedraogo, J. | Designing and Evaluating Scalable Child Marriage Prevention Programs in Burkina Faso and Tanzania: A Quasi-Experiment and Costing Study | 2020 | Burkina Faso and Tanzania | Girls aged 12 to 17 years | **Child marriage:** ever been married or lived with a person as married | Multiple interventions; Community dialogues; Intervention with traditional/religious leaders (Tanzania); Life skills (Tanzania) |  |
| Chow, V., & Vivalt, E. | Challenges in Changing Social Norms: Evidence from Interventions Targeting Child Marriage in Ethiopia | 2022 | Ethiopia | Girls aged 8-17 years | **Child marriage:** ever married | Multiple interventions; Community dialogues; Life skills; Intervention with traditional/religious leaders |  |
