# Supplementary material for: Scoping review of social norms interventions to reduce violence and improve SRHR outcomes among adolescents and young people in sub-Saharan Africa
Source: Front Reprod Health. 2025 May 15;7:1592696. doi: 10.3389/frph.2025.1592696 (PMC12119623; doi:10.3389/frph.2025.1592696)
Supplement: Supplementary file 3 [file Table3.docx]

|  | **Change Social Expectations** | | | | | **Publicise & diffuse** | | **Catalyse & reinforce** | | |
| --- | --- | --- | --- | --- | --- | --- | --- | --- | --- | --- |
| **Attributes** | 1. Has accurate understanding of existing social norms, power dynamics and reference groups | 2. Targets pluralistic ignorance where there is a discrepancy between the actual norm and what people think others expect of them | 3. Promotes a positive new norm or associated behaviour and/or seeks to weaken existing harmful norms by correcting associated knowledge | 4. Engages at multiple levels of the socio-ecological framework including by creating spaces for family and community and institutional change | 5. Promotes commitments to community change | 6. Has a diffusion strategy beyond individual behaviour change | 7. Publicises role models and benefits of new behaviour and /or avoids reinforcing negative norms and behaviours. | 8. Creates new rewards and sanctions, including legal and policy change | 9. Reinforce opportunities to behave in accordance with new norm | 10. Addresses other institutional or individual factors that promote risk behaviours and impede access to SRH services |
| **Criteria** | Uses formative/participatory research with intervention communities to understand local contexts or engage them in intervention design, fully articulating what the norms, power dynamics and reference groups are | Presents information on the prevalence of norms or behaviours to participants, including through mass media, social marketing campaigns or small group workshops in which the group norm is assessed and the misperception is discussed | Emphasizes positive norms and related behaviours such as healthy relationships, gender empowerment, non-violent relationships, positive communication, use of SRHR services  Presents information to change harmful beliefs, including in relation to gender inequitable attitudes, stigma and harmful cultural/community beliefs | Includes tailored intervention messaging, content, or design with reference groups beyond young people in mind | Prompts community members to publicly commit to change using pacts, pledges, action plans, goal setting | Encourages participants to share learnings with peers, family, and community members to raise awareness  Sparks critical reflection to shift norms first within a core group, who then engage others to have community-level impact.  May include promoting social movements to diffuse change. May include mass media, but only applies when media is used to publicize change in behaviours/norms in participants in the intervention.  Has a strategy to track diffusion. | Publicises role models including intervention participants, community members, peer educators or 'digital role models’ and their positive behaviours | Changes in legal framework to reward or sanction behaviours, or advocates for legal/policy change with institutions | Provides availability of SRH services encouraged in intervention, or opportunities to apply new knowledge in group discussions or through intervention, clearly articulating these as opportunities to practice behaviours | Provides socioeconomic support, health services, or other services that address other barriers to behavioural change. |

**Supplementary Table 3:** Criteria for attribute assessment
